# Proteomics profiling of research models for studying pancreatic ductal adenocarcinoma

**DOI:** 10.1038/s41597-025-04522-x

**Published:** 2025-02-14

**Authors:** Mathilde Resell, Hanne-Line Rabben, Animesh Sharma, Lars Hagen, Linh Hoang, Nan T. Skogaker, Anne Aarvik, Eirik Knudsen Bjåstad, Magnus K. Svensson, Manoj Amrutkar, Caroline S. Verbeke, Surinder K. Batra, Gunnar Qvigstad, Timothy C. Wang, Anil Rustgi, Duan Chen, Chun-Mei Zhao

**Affiliations:** 1 Department of Clinical and Molecular Medicine, Norwegian University of Science and Technology, Trondheim, Norway; 2 PROMEC - Proteomics and Modomics Experimental Core Facility at NTNU and the Central Norway Regional Health Authority, Trondheim, Norway; 3 Department of Pathology, Oslo University Hospital, Oslo, Norway; 4 Institute of Clinical Medicine, University of Oslo, Oslo, Norway; 5 Department of Biochemistry and Molecular Biology, University of Nebraska College of Medicine, Nebraska, USA; 6 Department of Gastroenterology, St.Olav’s Hospital, Trondheim, Norway; 7 Division of Digestive and Liver Diseases, Herbert Irving Comprehensive Cancer Center, Columbia University Irving Medical Center, New York, USA

**Keywords:** Pancreatic cancer, Diseases, Cancer

## Abstract

Pancreatic ductal adenocarcinoma (PDAC) remains one of the most lethal malignancies, with a five-year survival rate of 10–15% due to late-stage diagnosis and limited efficacy of existing treatments. This study utilized proteomics-based systems modelling to generate multimodal datasets from various research models, including PDAC cells, spheroids, organoids, and tissues derived from murine and human samples. Identical mass spectrometry-based proteomics was applied across the different models. The preparation and validation of the research models and the proteomics were described in detail. The assembly datasets we present here contribute to the data collection on PDAC, which will be useful for systems modelling, data mining, knowledge discovery in databases, and bioinformatics of individual models. Further data analysis may lead to the generation of research hypotheses, predictions of targets for diagnosis and treatment, and relationships between data variables.

## Background & Summary

Pancreatic ductal adenocarcinoma (PDAC) constitutes >90% of all pancreatic cancer cases^1^. It is one of the deadliest forms of cancer, with a five-year survival rate of 10–15% and a median survival of 4.6 months^2,3^. PDAC is often at an advanced stage at the time of clinical detection and is resistant to treatments that are highly effective for other types of cancer, including chemotherapy, radiotherapy, immunotherapy, and targeted therapies^4^. The reasons of the difficulty in treating patients with PDAC are still unclear concerning molecular mechanisms^5,6^. Thus, it is urgently needed to accelerate the progress of translational research or translational medicine of PDAC, i.e., “from bench to bedside” in the identification of biomarkers for the development of effective treatments.

Transcriptomics has been widely applied for studying many cancer types, but is still difficult to study the pancreas, mainly because of the difficulty in the preparation of high-quality of RNA due to the high level of ribonuclease and protease digestive enzymes in acinar cells. Of note, this difficulty is particularly considered when using normal pancreatic tissue as a control in studying PDAC. In this work, we have performed “qualitative proteomics” and identified proteins (with relative intensities) in biological samples collected from various research models that have been applied for studying PDAC. It is not uncommon that there are similarities and differences between the models, and it is common that selections of different research models are dependent on research purposes. A significant obstacle in the translational medicine of PDAC is the discrepancies that exist between research models and patients. To understand and address this challenge, we present assembly datasets of proteomics across the different research models and patients. The research models included cells, spheroids, organoids, and tissue from murine and human samples with detailed information on model preparations, proteomics protocols, and technical analysis in connection with the quality of the measurements, data records, and code availability.

**Fig. 1:**
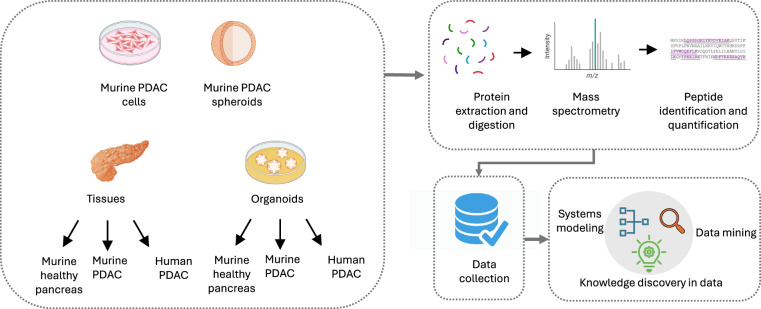
Study design showing research models, proteomic analysis, and utilization. The workflow includes protein samples from the different research models for protein extraction and digestion followed by mass spectrometry for peptide identification and quantification. The raw data is pooled for data collection and used for systems modelling, data mining, and knowledge discovery in data.

The datasets presented here will allow for comparisons of the similarities and differences between the research models and human PDAC tissue, identifying potential matches and mismatches. For instance, the comparisons by systems modelling will help select which method most closely resemble human PDAC tissue, guiding future research and enhancing the development of more effective PDAC therapies. The datasets presented here can also be leveraged in multiple ways, including the identification of new drug targets by analysing crucial proteins and signalling pathways, better understanding of disease mechanisms, discovery of biomarkers for treatment monitoring, development of treatment strategies targeting multiple aspects of PDAC, and validation of representability of various research models. The data collection (particularly being further enriched) will also be useful for data mining (e.g., clustering or segmentation to group similar data items into subsets) and knowledge discovery in data (e.g., joint probability density function of variables that have a causal or deterministic relationship). These datasets might also be useful for bioinformatics of individual models. By making these datasets publicly accessible, the research community can collectively enhance the understanding of PDAC and accelerate translational research between laboratory research and clinical trials, which complies with the European Health Data Space (EHDS), given that it provides a consistent, trustworthy, and efficient system for reusing health data for research, innovation, policy-making, and regulatory activities in general (Fig. 1).

## Methods

### Ethics

All procedures involving human participants and animals were performed in accordance with the ethical standards of the institutional and/or national research committee and the Helsinki Declaration and its later amendments or comparable ethical standards (for humans: REK South-east 2015/738; for animals: Norwegian Food Safety Authority Mattilsynet FOTS 8335). The human tumour tissues were samples of Pancreatic Biobank Oslo University Hospital (OUH).

### Sample collection

Thirty biological samples were collected from eight research models (Table 1). The sample numbers were six in the cells, murine PDAC tumour tissues and murine normal pancreatic tissues; three in murine PDAC spheroids, human PDAC organoids and human PDAC tissues; two in murine pancreatic exocrine organoids; and one in murine PDAC organoids. Of note, it was estimated that the numbers of organoids per model was 2000–6000. The sample sizes were chosen based on resources we have had during the study period. Six PDAC mice and six normal mice were used in consideration of the 95% confidence limit in case of “quantitative proteomics” is needed. (The relative intensities of proteins are available in Data Records).

**Table 1:** Overview of research models.

Research model	Description
Murine PDAC cells	UN-KC-6141 cell line derived from C57BL/6 mice with KrasG12D; Pdx1-Cre mutation
Murine PDAC tumour tissues	C57BL/6 mice
Murine normal pancreatic tissues	C57BL/6 mice
Murine PDAC spheroids	Derived from UN-KC-6141 cell line
Murine PDAC organoids	Derived from C57BL/6 mice
Murine pancreatic exocrine organoids	Derived from C57BL/6 mice (Stem Cell)
Human PDAC organoids	Derived from a patient (with tumour >3 cm)
Human PDAC tumour tissues	Patients (with tumour >2–3 cm)

### Preparation of research models

#### Murine PDAC cell line

The UN-KC-6141 cell line originates from a pancreatic tumour in a genetically modified mouse that was designed to mimic human PDAC. This mouse, carrying the KrasG12D;Pdx1-Cre mutation, was 50 weeks old when the tumour was harvested. The culture procedure included subculturing and passaging with required reagents, materials and equipment for handling to ensure that the cell line retains crucial genetic features and characteristics (see Supplementary materials under “Murine PDAC cell culture procedure”). It should be noticed that this cell line has been publicly available since the original article was published^7^ and widely used in different laboratories^8,9^.

#### Murine PDAC spheroids

The PDAC spheroids were generated using the aforementioned cells, employing the same reagents and cultivation methods. The spheroids were formed by using the hanging drop method, and passage 39 was used for this purpose. The drops were placed into the underside of the lid of a petri dish and subsequently inverted so that each drop was held in place by surface tension and gravity. The droplets consisted of 60 000 cells/27 µl and the dishes were filled with 1 ml medium to avoid evaporation and provide nutrition. All samples were cultured and kept in a humidified incubator of 37 °C and 5% CO_2_. The spheroids were collected for proteomic analysis after 7 days. The formation procedure included the preparation of media, cultivation, and passage (see Supplementary materials under “Murine PDAC spheroid formation procedure”).

#### Murine PDAC organoids and normal mouse organoids of exocrine pancreas

The PDAC organoids were originally generated at Cold Spring Harbor Laboratory by isolating PDAC tissue from C57BL/6 mice according to the Tuveson Laboratory Murine and Human Organoid Protocols (http://tuvesonlab.labsites.cshl.edu/wp-content/uploads/sites/49/2018/06/20170523_OrganoidProtocols.pdf). The normal pancreatic exocrine organoids used were obtained from Stem Cell Technologies. The murine organoids grow faster than human organoids, necessitating a higher splitting ratio during passaging. Split ratios ranged from 1:4 to 1:8, based on the confluency of the organoids^10^. The same cultivation protocol was used for both PDAC and normal organoids (see Supplementary materials under “Murine organoid cultivation procedure”).

#### Human PDAC organoids

The organoids were acquired from ATCC, derived from the primary pancreatic tissue obtained from the pancreatic head of a 61-year-old female. The patient, whose tissue was used to generate these organoids, was diagnosed with Stage III PDAC. Notably, the patient had not undergone any neoadjuvant therapy at the time of organoid derivation. Given that human organoids proliferate more slowly compared to murine organoids, and thrive better at higher densities, the splitting ratios were between 1:1 and 1:3. Rho kinase inhibitor was added upon thawing and during any stress-prone stages to enhance organoid viability by stabilizing the cytoskeleton and maintaining cellular interactions. The cultivation procedure included preparations of Wnt-3a conditioned medium and Rspondin-1 conditioned medium, cultivation, and passage (see Supplementary materials under “Human PDAC organoid cultivation procedure”).

#### Mouse PDAC tumour tissues and mouse normal pancreatic tissues

Both male and female mice (C57/BL6, 5–8 weeks old) were injected with the murine PDAC cells into the pancreas. The number of injected cells was 2.5 × 10^5^ and cell passage was 32. Age-, sex- and genetically matched normal mice were included. Both PDAC tumour tissue and normal pancreatic tissue were collected. The animal experiment protocol included equipment, procedure of cell transplantation, and collection of tissue samples (see Supplementary materials under “Age-matched normal mouse tissue and PDAC mouse tissue preparations”).

### Tumour tissue of PDAC patients

Surgical biopsies were taken from three patients (Table 1), including tissue cylinders measuring 2 mm in diameter and up to a max 10 mm in length, and the cylindered sample was cut into a minimum of 5 pieces before fixation for electron microscopy. Each tissue sample was embedded in one resin block after processing. In addition, two biopsy cores were taken that produced tissue cylinders measuring 3 mm in diameter and up to 10 mm in length. The samples were kept in a nitrogen tank for proteomics.

### Morphology

Assessment at the level of light microscopy was performed by staining with hematoxylin & eosin (H&E) and toluidine blue (TBO) which is a basic thiazine metachromatic dye with high affinity for acidic tissue components (e.g., DNA and RNA) and immunohistochemistry (IHC). Assessment at the ultrastructural level was performed by staining with alcoholic uranyl acetate and lead citrate (UA-LC) for transmission electron microscopy (TEM) and/or with sputter-coated gold/palladium for scanning electron microscopy (SEM) (for the analysis preparations, see supplementary materials under “Electron microscopy”).

### Proteomics

#### Equipment and procedure

Samples were added 450 μl 100 mM Tris-HCl pH- 8.5 and homogenized using an ultra-turrax (IKA) (this step was omitted for cell experiments). The buffer was adjusted to final 1% SDC, 100 mM Tris-HCl pH- 8.5, 10 mM TCEP, 40 mM CAA, and sonicated 10 cycles (30 sec ON/30 sec OFF) using the Bioruptor® Pico sonicator, and further heated at 95 °C for 10 min. Debris was spun down (16000*g 10 min) and 50 μg soluble protein of each sample was added 100 μl 0.1 M Ammonium bicarbonate, 1 μg trypsin, and digested over-night at 37 °C. Peptides were desalted using C18 spin columns, dried in a speedVac^TM^ centrifuge, and resuspended in 0.1% Formic acid before MS analysis. LC-MS/MS was performed on a timsTOF Pro (Bruker Daltonics) connected to a nanoElute (Bruker Daltonics) HPLC. Peptide separation was done using a PepSep 25 (150um*25 cm) column with running buffers A (0.1% formic acid) and B (0.1% formic acid in acetonitrile) with a gradient from 2% B to 40 for 100 min. The timsTOF instrument was operated in the DDA PASEF mode with 10 PASEF scans per acquisition cycle and accumulation and ramp times of 100 ms each. The ‘target value’ was set to 20,000 and dynamic exclusion was activated and set to 0.4 min. The quadrupole isolation width was set to 2 Th for m/z < 700 and 3 Th for m/z > 800.” Of note, the procedures from sample preparation to MS were identical for all models and patients, except that the homogenization was not needed for the cell experiments. All raw data were collected and technically controlled, and no technical replicates were performed. (see Supplementary materials under “Proteomics procedure”).

#### Data analysis

MaxQuant was used to analyse the mass spectrometry (MS) data by identifying and quantifying proteins in the biological samples. The peptides are identified with the Andromeda search engine and quantified proteins using label-free quantification (LFQ). MaxLFQ, a feature of MaxQuant, uses advanced algorithms to determine protein abundances by extracting and normalizing peptide signals across multiple samples. The software aligns retention times and matches features between runs, ensuring accurate and robust protein quantification. MaxQuant’s user-friendly interface and integration with statistical tools make it ideal for proteomics research^11^. The false discovery rate was kept at 1% and only unique peptides with high confidence were used for final protein group identification.

## Data Records

The mass spectrometry proteomics raw data have been deposited to the ProteomeXchange Consortium via the PRIDE [ref PubMed ID: 34723319] partner repository with the dataset on PRIDE-links (Table 2).

**Table 2:** Research models with PRIDE-links.

Research model	PRIDE-link
Murine PDAC cells	https://identifiers.org/pride.project:PXD057793^13^
Murine PDAC tumour tissues	https://identifiers.org/pride.project:PXD057795^14^
Murine normal pancreatic tissues	https://identifiers.org/pride.project:PXD057798^15^
Murine PDAC spheroids	https://identifiers.org/pride.project:PXD057804^16^
Murine PDAC organoids	https://identifiers.org/pride.project:PXD057888^17^
Murine pancreatic exocrine organoids	https://identifiers.org/pride.project:PXD057829^18^
Human PDAC organoids	https://identifiers.org/pride.project:PXD057928^19^
Human PDAC tumour tissues	https://identifiers.org/pride.project:PXD057607^20^

(For detailed information, see Supplementary materials under “Table S1: Data records for navigating the repository”, and Table S1. xlsx)

## Technical Validation

### Protein identification

The total numbers of detected proteins in the research models and patients were in the range between 2723 and 5800 in terms of uniport ID which corresponded to 2698 and 5726 in terms of gene name (Table 3). [for the proteins (names and intensity), see Data Records above].

**Table 3:** Number of identified proteins in research models and patients.

Research model	# of proteins (uniport ID)	# of proteins (gene name)
Murine PDAC cells	4880	4813
Murine PDAC tumour tissues	4696	4652
Murine normal pancreatic tissues	2723	2698
Murine PDAC spheroids	3671	3650
Murine PDAC organoids	5800	5726
Murine pancreatic exocrine organoids	5421	5362
Human PDAC organoids	5306	5221
Human PDAC tumour tissues	3872	3765

### Quality of measurements

#### Murine PDAC cells

**Fig. 2:**
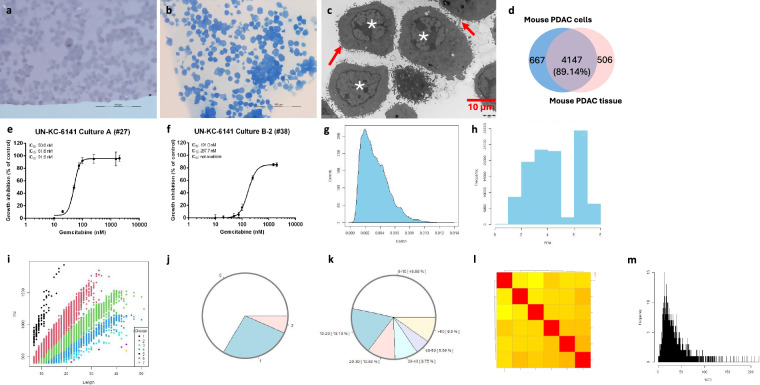
Quality measurements of murine PDAC cells. Of note, photomicrographs of cells stained with H&E (**a**) and TBO (**b**) and with alcoholic uranyl acetate and lead citrate for TEM (**c**) showing a clustered and pavement-like appearance and abnormal nuclear structure (indicated in asterisks in c) with microvilli on the plasma membrane (red arrows in c). Nearly 90% proteins of the cells are identical to the PDAC tumours that were formed from the cells (**d**). Functionally, the cells were able to develop a chemo-drug resistance (from passage #27 with IC_50_ at 50.0 nM to #38 with IC_50_ at 191.0 nM), a characteristic of PDAC (e and f), thereby offering a valuable model for preclinical studies and therapeutic testing^7^. The distribution of dmass for peptide ions with the peak is close to zero (0.002), indicating a high accuracy (**g**). The distribution of mass error ppm is somehow in a diverse range between 1 and 8 (**h**). The graph depicting peptide length versus m/z displays a positive correlation, which was expected as longer peptides typically have greater masses and higher mass-to-charge ratios (**i**). The missed cleavage chart indicates efficient enzymatic digestion with a predominant proportion of peptides exhibiting ‘0’0 missed cleavages (**j**), which could be due to in-solution digestion^12^. The sequence coverage pie chart shows predominant portions of proteins with 0–10 in 46.58% and 10–20 in 18.19% sequence coverage (**k**). Heatmap shows a positive correlation in log_2_ transformed intensity values between the six samples [Pearson’s correlation coefficient (**r**) showed in legend] (**l**). Histogram of protein group intensity shows the peak of coefficient of variation (CV) in % versus frequency distribution being less than 25% (**m**).

The cells (6–10 × 10^6^ per flask) in culture maintained the epithelial characteristics, such as polygonal in shape and growth in monolayer attached to the surface. Quality measurements showed the morphology by H&E, TB, TEM, and SEM, functions in terms of proteins and cell growth in response to chemotherapy. MS data analysis showed the distribution of mass tolerance distribution (dmass) for precursor ions and the distribution of mass error in parts per million (ppm), peptide length distribution, missed cleavage distribution, protein coverage distribution, heatmap between the six samples and histogram of protein group intensity (Fig. 2).

#### Murine PDAC tumour tissues

**Fig. 3:**
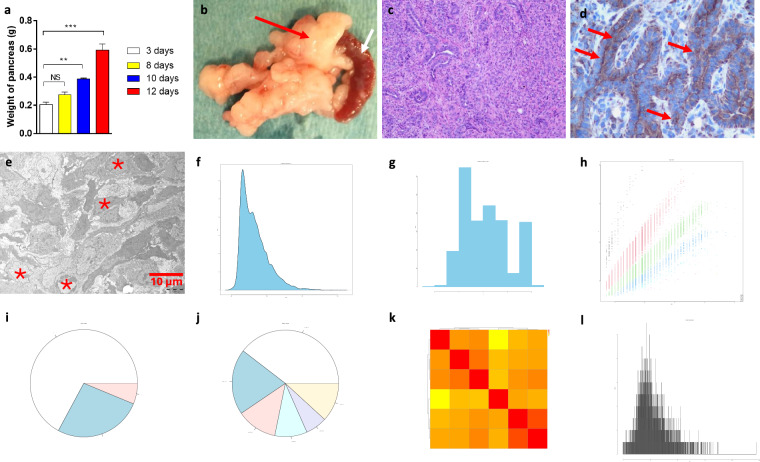
Quality measurements of murine PDAC tumours. Note, the tumour formation and growth during a period from 3 to 12 days [mean + SEM (n = 3). NS: not significant; **^,^ ***p < 0.01, 0.001 (one-way ANOVA with Dunnett’s post hoc test] (**a**), representative photomicrograph of tumour at 12 days (indicated by red arrow) (spleen which is connected with pancreas is indicated by white arrow)(**b**), photomicrographs of H&E (**c**) and IHC with antibody against epithelial cell marker cytokeratin 19 (red arrows) (**d**), and TEM (**e**). In e, the PDAC tumour tissue displays high cellular density and abnormal nuclear structure, such as irregular profile, coarse heterochromatin aggregates, enlarged nucleoli, and less lamina-associated heterochromatin (indicated in asterisks). The distribution of dmass for peptide ions with the peak is close to zero (0.002), indicating a high accuracy (**f**). The distribution of mass error in ppm is somehow in a diverse range between 1 and 8 (**g**). The graph depicting peptide length versus m/z displays a positive correlation, which was expected as longer peptides typically have greater masses and higher mass-to-charge ratios (**h**). The missed cleavage chart indicates efficient enzymatic digestion with a predominant proportion of peptides exhibiting ‘0’0 missed cleavages (**i**), which could be due to in-solution digestion^12^. The sequence coverage pie chart shows predominant portions of proteins with 0–10 in 39.57% and 10–20 in 19.92% sequence coverage (**j**). Heatmap shows a positive correlation in log_2_ transformed intensity values between the samples [Pearson’s correlation coefficient (**r**) showed in legend] (**k**). Histogram of protein group intensity shows the peak of coefficient of variation (CV) in % versus frequency distribution being less than 50% (**l**).

After the cells were orthotopically injected into the pancreas, PDAC tumour was formed, reflecting by increased weight of pancreas and neoplasia measured by histology and proteomics including the distribution of dmass for precursor ions and the distribution of mass error in ppm, peptide length distribution, missed cleavage distribution, protein coverage distribution, heatmap between the six samples and histogram of protein group intensity (Fig. 3).

#### Murine normal pancreatic tissues

**Fig. 4:**
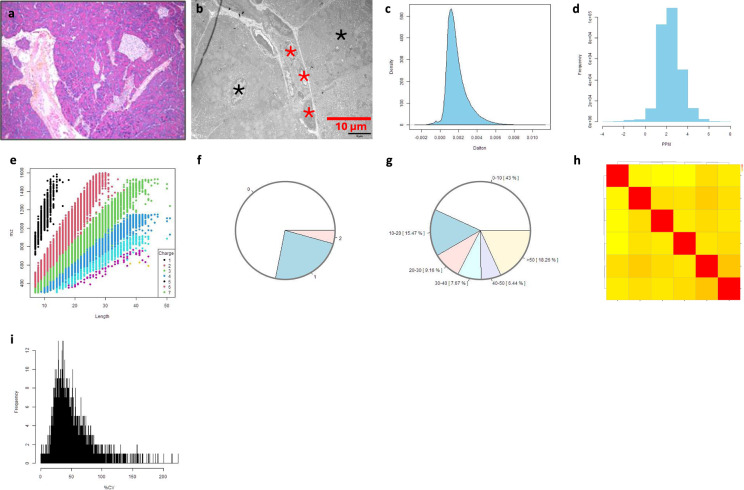
Quality measurements of murine normal pancreas. Note: representative photomicrographs of H&E (**a**) and TEM (**b**) of the pancreas with exocrine cells and ductal cells (indicated in red asterisks) and acinar cells (black asterisks) in B. The distribution of dmass for peptide ions with the peak is close to zero (0.002), indicating a high accuracy (**c**). The distribution of mass error in ppm is somehow in a diverse range between 1 and 6 (**d**). The graph depicting peptide length versus m/z displays a positive correlation, which was expected as longer peptides typically have greater masses and higher mass-to-charge ratios (**e**). The missed cleavage chart indicates efficient enzymatic digestion, with a predominant proportion of peptides exhibiting ‘0’0 missed cleavages (**f**), which could be due to in-solution digestion^12^. The sequence coverage pie chart shows predominant portions of proteins with 0–10 in 43% (**g**). Heatmap shows a positive correlation in log_2_ transformed intensity values between the six samples [Pearson’s correlation coefficient (**r**) showed in legend] (**h**). Histogram of protein group intensity shows the peak of coefficient of variation (CV) in % versus frequency distribution being about 25% (**i**).

Morphology at both light microscopy and TEM showed a normal appearance of the pancreas, particularly the exocrine part of the pancreas. MS data analysis showed the distribution of dmass for precursor ions and the distribution of mass error in ppm, peptide length distribution, missed cleavage distribution, protein coverage distribution, heatmap between the samples and histogram of protein group intensity (Fig. 4).

#### Murine PDAC spheroids

**Fig. 5:**
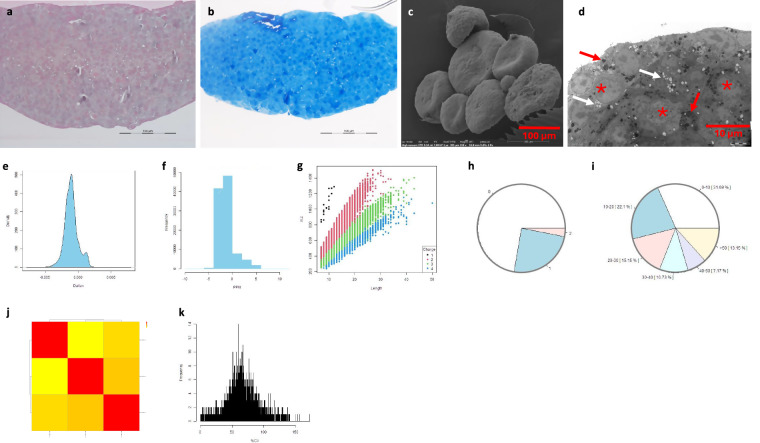
Quality measurements of murine PDAC spheroids. Note: representative photomicrographs of a spheroid stained with H&E (**a**) and TBO (**b**) and visualized with SEM (**c**) and TEM (**d**). Of note, the spheroid is almost without extracellular space/matrix (A and B) and exhibits a moon-like surface (**c**) and cells containing abnormal nuclear structure (indicated by asterisks) and vacuoles (white arrows) and lipofuscin bodies (red arrows) in the cytoplasm (**d**). The distribution of dmass for peptide ions with the peak is close to zero (0.002), indicating a high accuracy (**e**). The distribution of mass error in ppm is close to zero (**f**). The graph depicting peptide length versus m/z displays a positive correlation, which was expected as longer peptides typically have greater masses and higher mass-to-charge ratios (**g**). The missed cleavage chart indicates efficient enzymatic digestion, with a predominant proportion of peptides exhibiting ‘0’0 missed cleavages (**h**), which could be due to in-solution digestion^12^. The sequence coverage pie chart shows predominant portions of proteins with 0–10 in 31.69% (**i**). Heatmap shows a potential positive correlation in log_2_ transformed intensity values between the three samples [Pearson’s correlation coefficient (**r**) showed in legend] (**j**). Histogram of protein group intensity shows the peak of coefficient of variation (CV) in % versus frequency distribution being about 60% (**k**).

Morphology at both light microscopy and SEM/TEM showed PDAC spheroid displaying compacted tissue structure. MS data analysis showed the distribution of dmass for precursor ions and the distribution of mass error in ppm, peptide length distribution, missed cleavage distribution, protein coverage distribution, heatmap between the samples and histogram of protein group intensity (Fig. 5).

#### Murine PDAC organoids

**Fig. 6:**
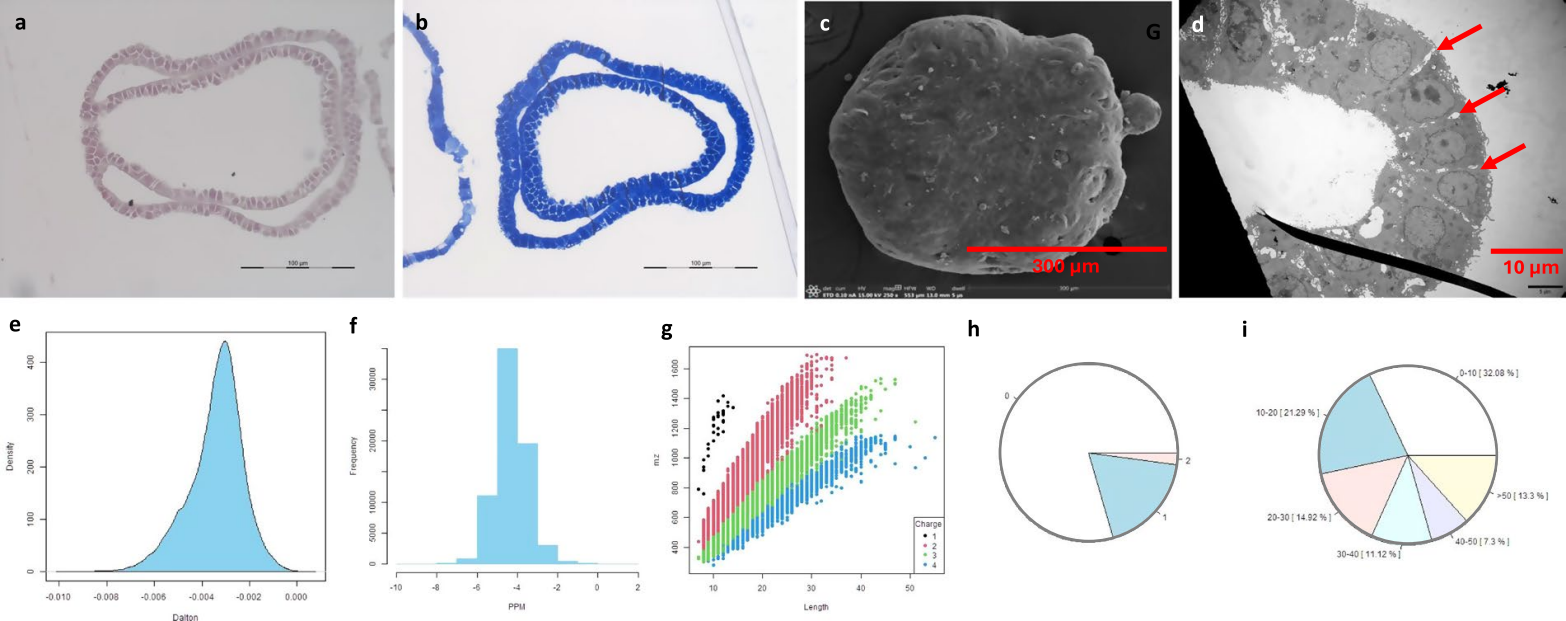
Quality measurements of murine PDAC organoids. Note: photomicrographs of an organoid stained with H&E (**a**) and TBO (**b**) and visualized with SEM (**c**) and TEM (**d**). Of note, the organoid exhibits a single layer of cells (folded in **a** and **b**) with a moon-like surface (**c**) and extracellular matrix between cells (arrows)(**d**). The distribution of dmass for peptide ions with the peak is close to zero (≈ -0.003), indicating a high accuracy (**e**). The distribution of mass error in ppm is close to -5 (**f**). The graph depicting peptide length versus m/z displays a positive correlation, which was expected as longer peptides typically have greater masses and higher mass-to-charge ratios (**g**). The missed cleavage chart indicates efficient enzymatic digestion, with a predominant proportion of peptides exhibiting ‘0’0 missed cleavages (**h**), which could be due to in-solution digestion^12^. The sequence coverage pie chart shows predominant portions of proteins with 0–10 in 32.08% (**i**).

Morphology at both light microscopy and SEM/TEM showed PDAC organoids. MS data analysis showed the distribution of dmass for precursor ions and the distribution of mass error in ppm, peptide length distribution, missed cleavage distribution, protein coverage distribution and histogram of protein group intensity (Fig. 6).

#### Murine pancreatic exocrine organoids

**Fig. 7:**
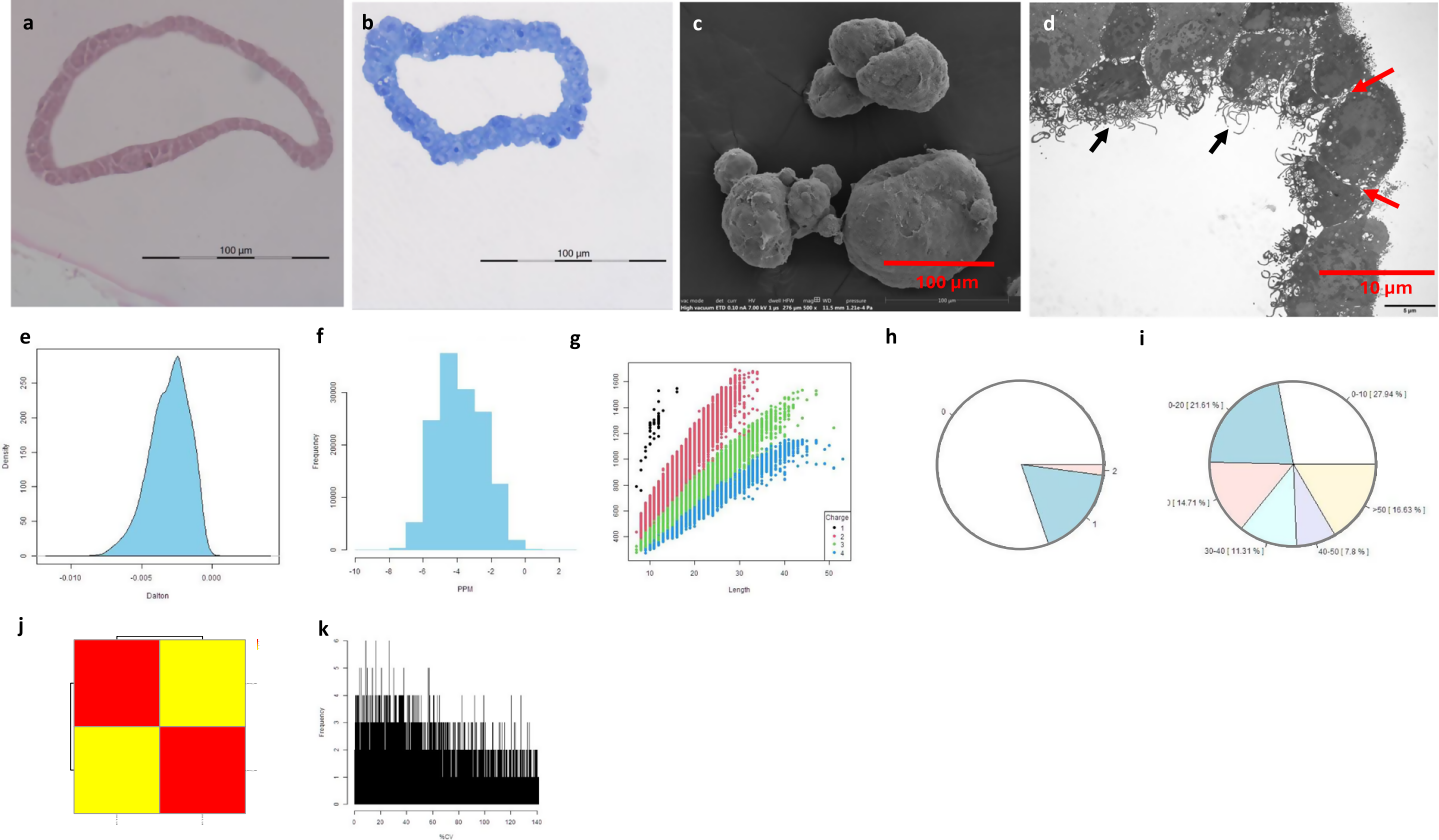
Quality measurements of murine normal exocrine organoids. Note: photomicrographs of an organoid stained with H&E (**a**) and TBO (**b**) and visualized with SEM (**c**) and TEM (**d**). Of note, the organoid exhibits a single layer of cells (**a** and **b**) with the moon-like surface (**c**) and microvilli (black arrows) and extracellular matrix (red arrows) between cells (arrows)(**d**). The distribution of dmass for peptide ions with the peak is close to zero (≈ -0.002), indicating a high accuracy (**e**). The distribution of mass error in ppm is close to -4 (**f**). The graph depicting peptide length versus m/z displays a positive correlation, which was expected as longer peptides typically have greater masses and higher mass-to-charge ratios (**g**). The missed cleavage chart indicates efficient enzymatic digestion, with a predominant proportion of peptides exhibiting ‘0’0 missed cleavages (**h**), which could be due to in-solution digestion^12^. The sequence coverage pie chart shows predominant portions of proteins with 0–10 in 27.94% (**i**). The heatmap shows a correlation in log_2_ transformed intensity values between the two samples [Pearson’s correlation coefficient (**r**) showed in legend] (**j**). Histogram of protein group intensity shows no peak of coefficient of variation (CV) in % versus frequency distribution (**k**).

Morphology at both light microscopy and SEM/TEM showed normal pancreatic exocrine organoids. MS data analysis showed the distribution of dmass for precursor ions and the distribution of mass error in ppm, peptide length distribution, missed cleavage distribution, protein coverage distribution, heatmap between the samples and histogram of protein group intensity (Fig. 7).

#### Human PDAC organoids

**Fig. 8:**
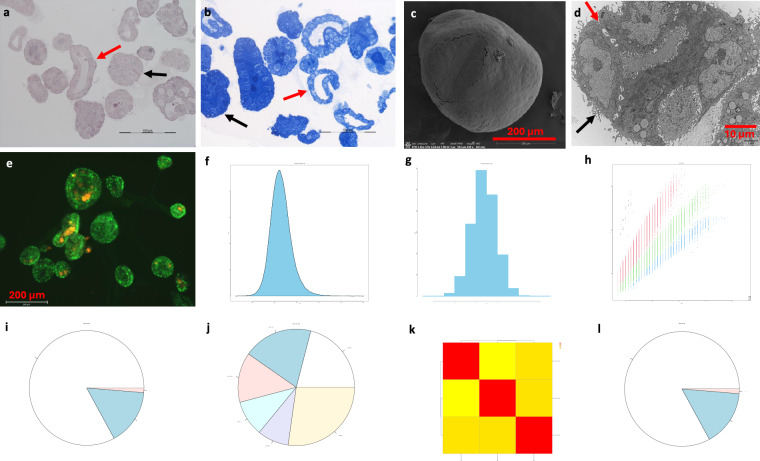
Quality measurements of human PDAC organoids. Note: photomicrographs of organoids stained with H&E (**a**) and TBO (**b**) and visualized with SEM (**c**) and TEM (**d**). Of note, the organoid exhibits either a single layer of cells (red arrows) or cluster-like cells (black arrows) (**a** and **b**) with the moon-like surface (**c**) and microvilli (black arrow) and extracellular matrix (red arrow) between cells (**d**). Cell image analysis (with EVOS M7000) shows the organoids in various sizes in profile with high proliferative activity (IHC with antibody to KI67 in green colour) and low apoptotic activity (IHC with antibody to caspase 3 in orange colour) (**e**). The distribution of dmass for peptide ions with the peak is close to zero (≈ 0.001), indicating a high accuracy (**f**). The distribution of mass error in ppm is close to 1 (**g**). The graph depicting peptide length versus m/z displays a positive correlation, which was expected as longer peptides typically have greater masses and higher mass-to-charge ratios (**h**). The missed cleavage chart indicates efficient enzymatic digestion, with a predominant proportion of peptides exhibiting ‘0’0 missed cleavages (**i**), which could be due to in-solution digestion^12^. The sequence coverage pie chart shows predominant portions of proteins with 0–10 in 20.98% (**j**). Heatmap shows a potential positive correlation in log_2_ transformed intensity values between the three samples [Pearson’s correlation coefficient (**r**) showed in legend] (**k**). Histogram of protein group intensity shows the peak of coefficient of variation (CV) in % versus frequency distribution being about 25% (**l**).

Morphology at both light microscopy and SEM/TEM showed PDAC organoids. MS data analysis showed the distribution of dmass for precursor ions and the distribution of mass error in ppm, peptide length distribution, missed cleavage distribution, protein coverage distribution, heatmap between the samples and histogram of protein group intensity (Fig. 8).

#### Human PDAC tumor tissues

**Fig. 9:**
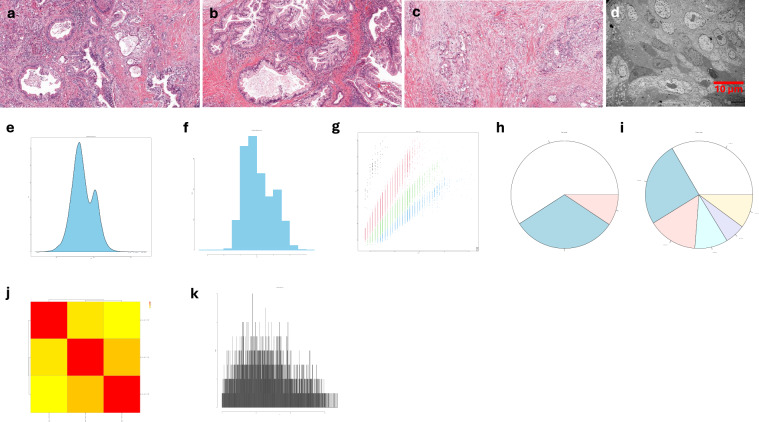
Quality measurements of human PDAC tumour tissues. Note: photomicrographs of tissues stained with H&E in a 69-year-old male patient who had a large tumour > 3 cm (T3) that has spread to 2 lymph nodes (N2) but not spread to anywhere else in the body (MO) (**a**), 79-year-old male patient with T2N0M0 (**b**) and 75-year-old male patient with T3N2M0 (**c**). TEM shows PDAC cells that are similar to those of murine PDAC cells, murine PDAC tumour, murine PDAC spheroids and organoids, and human PDAC organoids (**d**). The distribution of dmass for peptide ions with the peak is close to zero indicates a high accuracy (**e**). The distribution of mass error in ppm is close to -3 (**f**). The graph depicting peptide length versus m/z displays a positive correlation, which was expected as longer peptides typically have greater masses and higher mass-to-charge ratios (**g**). The missed cleavage chart indicates efficient enzymatic digestion, with a predominant proportion of peptides exhibiting ‘0’0 missed cleavages (**h**), which could be due to in-solution digestion^12^. The sequence coverage pie chart shows predominant portions of proteins with 0–10 in 33.37% (**i**). Heatmap shows a positive correlation in log_2_ transformed intensity values between the samples [Pearson’s correlation coefficient (**r**) showed in legend] (**j**). Histogram of protein group intensity shows the coefficient of variation (CV) in % versus frequency distribution being about 25%–100% (**k**).

The three patients were selected because of typical PDAC histological, cytological, and ultrastructural features with similar grades of differentiation. MS data analysis showed the distribution of dmass for precursor ions and the distribution of mass error in ppm, peptide length distribution, missed cleavage distribution, protein coverage distribution, heatmap between the samples and histogram of protein group intensity (Fig. 9).

## Supplementary information

Supplementary materials

## Data Availability

Data preparation and analysis procedures have been described in detail in the Methods section and the supplementary materials. MaxQuant^11^ version 2.3.1.0 was used for analysis and the code for generating figures using the Maxquant output is available at Github (https://row.githubusercontent.com/animesh/scripts/master/proteinGroupsQC.r and https://row.githubusercontent.com/animesh/scripts/master/evidenceQC.r).
