# Supplementary material for: Proteomics profiling of research models for studying pancreatic ductal adenocarcinoma
Source: Sci Data. 2025 Feb 14;12:266. doi: 10.1038/s41597-025-04522-x (PMC11828881; doi:10.1038/s41597-025-04522-x)
Supplement: Supplementary file 1 — Supplementary materials [file 41597_2025_4522_MOESM1_ESM.pdf]

Proteomics profiling of research models for studying pancreatic ductal adenocarcinoma

Mathilde Resell[1], Hanne-Line Rabben[1], Animesh Sharma[2], Lars Hagen[2], Linh Hoang[1], Nan T. Skogaker[1], Anne Aarvik[1], Eirik Knudsen Bjåstad[1], Magnus K. Svensson[1], Manoj Amrutkar[3], Caroline S. Verbeke[3,4], Surinder K. Batra[5], Gunnar Qvigstad[6], Timothy C. Wang[7], Anil Rustgi[7], Duan Chen[1], Chun-Mei Zhao[1*]

[1]Department of Clinical and Molecular Medicine, Norwegian University of Science and Technology, Trondheim, Norway; [2]PROMEC - Proteomics and Modomics Experimental Core Facility at NTNU and the Central Norway Regional Health Authority, Trondheim, Norway ;[3]Department of Pathology, Oslo University Hospital, Oslo, Norway; [3]Institute of Clinical Medicine, University of Oslo, Oslo, Norway; [4]Department Biochemistry and Molecular Biology, University of Nebraska College of Medicine, Nebraska, USA; [5]Department of Gastroenterology, St.Olav's Hospital, Trondheim, Norway; [6]Divison of Digestive and Liver Diseases, Herbert Irving Comprehensive Cancer Center, Columbia University Irving Medical Center, New York, USA

*Corresponding author: Chun-Mei Zhao (chun-mei.zhao@ntnu.no)

Supplementary materials

Table of Contents:

1. Table S1: Data records for navigating the repository
2. Murine PDAC cell culture procedure
3. Murine PDAC spheroid formation procedure
4. Murine organoid cultivation procedure
5. Human PDAC organoid cultivation procedure
6. Murine model of PDAC with age-matched control preparations
7. Proteomics procedure
8. Electron microscopy procedure
9. References

1. **Table S1: Data records for navigating the repository**

Group	File name	Pride ID	QC-protein group code	QC evidence code
Murine PDAC cells	PD/USERS/Mei/2016-05_PancreaticCancer/QE/20160505_Cell_1.raw	PXD057793	https://raw.githubusercontent.com/animesh/scripts/a4df43cfd47ea6a38e031a4d375f363915e624f9/proteinGroupsQC.rq	https://raw.githubusercontent.com/animesh/scripts/1a3e42f058403a45703c613e7049e9d2a31ee05c/evidenceQC.r
Murine PDAC cells	PD/USERS/Mei/2016-05_PancreaticCancer/QE/20160505_Cell_2.raw	PXD057793	https://raw.githubusercontent.com/animesh/scripts/a4df43cfd47ea6a38e031a4d375f363915e624f9/proteinGroupsQC.r	https://raw.githubusercontent.com/animesh/scripts/1a3e42f058403a45703c613e7049e9d2a31ee05c/evidenceQC.r
Murine PDAC cells	PD/USERS/Mei/2016-05_PancreaticCancer/QE/20160505_Cell_3.raw	PXD057793	https://raw.githubusercontent.com/animesh/scripts/a4df43cfd47ea6a38e031a4d375f363915e624f9/proteinGroupsQC.r	https://raw.githubusercontent.com/animesh/scripts/1a3e42f058403a45703c613e7049e9d2a31ee05c/evidenceQC.r
Murine PDAC cells	PD/USERS/Mei/2016-05_PancreaticCancer/QE/20160505_Cell_4.raw	PXD057793	https://raw.githubusercontent.com/animesh/scripts/a4df43cfd47ea6a38e031a4d375f363915e624f9/proteinGroupsQC.r	https://raw.githubusercontent.com/animesh/scripts/1a3e42f058403a45703c613e7049e9d2a31ee05c/evidenceQC.r
Murine PDAC cells	PD/USERS/Mei/2016-05_PancreaticCancer/QE/20160505_Cell_5.raw	PXD057793	https://raw.githubusercontent.com/animesh/scripts/a4df43cfd47ea6a38e031a4d375f363915e624f9/proteinGroupsQC.r	https://raw.githubusercontent.com/animesh/scripts/1a3e42f058403a45703c613e7049e9d2a31ee05c/evidenceQC.r
Murine PDAC cells	PD/USERS/Mei/2016-05_PancreaticCancer/QE/20160505_Cell_6.raw	PXD057793	https://raw.githubusercontent.com/animesh/scripts/a4df43cfd47ea6a38e031a4d375f363915e624f9/proteinGroupsQC.r	https://raw.githubusercontent.com/animesh/scripts/1a3e42f058403a45703c613e7049e9d2a31ee05c/evidenceQC.r
Murine PDAC tumor	PD/USERS/Mei/2016-05_PancreaticCancer/QE/20160505_Tumor_1.raw	PXD057795	https://raw.githubusercontent.com/animesh/scripts/9f75cc83502d0c789c7d2703276db7ebfd842d90/proteinGroupsQC.r	https://raw.githubusercontent.com/animesh/scripts/5c24b581325b315616e03d25c582f99234c53971/evidenceQC.r

Murine PDAC tumor	PD/USERS/Mei/2016-05_PancreaticCancer/QE/20160505_Tumor_2.raw	PXD057795	https://raw.githubusercontent.com/animesh/scripts/9f75cc83502d0c789c7d2703276db7ebfd842d90/proteinGroupsQC.r	https://raw.githubusercontent.com/animesh/scripts/5c24b581325b315616e03d25c582f99234c53971/evidenceQC.r
Murine PDAC tumor	PD/USERS/Mei/2016-05_PancreaticCancer/QE/20160505_Tumor_3.raw	PXD057795	https://raw.githubusercontent.com/animesh/scripts/9f75cc83502d0c789c7d2703276db7ebfd842d90/proteinGroupsQC.r	https://raw.githubusercontent.com/animesh/scripts/5c24b581325b315616e03d25c582f99234c53971/evidenceQC.r
Murine PDAC tumor	PD/USERS/Mei/2016-05_PancreaticCancer/QE/20160505_Tumor_4.raw	PXD057795	https://raw.githubusercontent.com/animesh/scripts/9f75cc83502d0c789c7d2703276db7ebfd842d90/proteinGroupsQC.r	https://raw.githubusercontent.com/animesh/scripts/5c24b581325b315616e03d25c582f99234c53971/evidenceQC.r
Murine PDAC tumor	PD/USERS/Mei/2016-05_PancreaticCancer/QE/20160505_Tumor_5.raw	PXD057795	https://raw.githubusercontent.com/animesh/scripts/9f75cc83502d0c789c7d2703276db7ebfd842d90/proteinGroupsQC.r	https://raw.githubusercontent.com/animesh/scripts/5c24b581325b315616e03d25c582f99234c53971/evidenceQC.r
Murine PDAC tumor	PD/USERS/Mei/2016-05_PancreaticCancer/QE/20160505_Tumor_6.raw	PXD057795	https://raw.githubusercontent.com/animesh/scripts/9f75cc83502d0c789c7d2703276db7ebfd842d90/proteinGroupsQC.r	https://raw.githubusercontent.com/animesh/scripts/5c24b581325b315616e03d25c582f99234c53971/evidenceQC.r
Murine normal pancreatic tissue	PD/USERS/Mei/2017-08_PancreaticCancer/QE/20170901_55.raw	PXD057798	https://raw.githubusercontent.com/animesh/scripts/bdabc21a58bae5b1fe4eb3e6ada01a677eda2e20/proteinGroupsQC.r	https://raw.githubusercontent.com/animesh/scripts/7bd19ef074bfd97d04c55741d519be6460534558/evidenceQC.r
Murine normal pancreatic tissue	PD/USERS/Mei/2017-08_PancreaticCancer/QE/20170901_56.raw	PXD057798	https://raw.githubusercontent.com/animesh/scripts/bdabc21a58bae5b1fe4eb3e6ada01a677eda2e20/proteinGroupsQC.r	https://raw.githubusercontent.com/animesh/scripts/7bd19ef074bfd97d04c55741d519be6460534558/evidenceQC.r
Murine normal pancreatic tissue	PD/USERS/Mei/2017-08_PancreaticCancer/QE/20170901_57.raw	PXD057798	https://raw.githubusercontent.com/animesh/scripts/bdabc21a58bae5b1fe4eb3e6ada01a677eda2e20/proteinGroupsQC.r	https://raw.githubusercontent.com/animesh/scripts/7bd19ef074bfd97d04c55741d519be6460534558/evidenceQC.r
Murine normal	PD/USERS/Mei/2017-	PXD057798	https://raw.githubusercontent.com/animesh/scripts	https://raw.githubusercontent.com/animesh/scripts

pancreatic tissue	08_PancreaticCancer/QE/20170901_58.raw		/bdabc21a58bae5b1fe4eb3e6ada01a677eda2e20/proteinGroupsQC.r	/7bd19ef074bfd97d04c55741d519be6460534558/evidenceQC.r
Murine normal pancreatic tissue	PD/USERS/Mei/2017-08_PancreaticCancer/QE/20170901_59.raw	PXD057798	https://raw.githubusercontent.com/animesh/scripts/bdabc21a58bae5b1fe4eb3e6ada01a677eda2e20/proteinGroupsQC.r	https://raw.githubusercontent.com/animesh/scripts/7bd19ef074bfd97d04c55741d519be6460534558/evidenceQC.r
Murine normal pancreatic tissue	PD/USERS/Mei/2017-08_PancreaticCancer/QE/20170901_60.raw	PXD057798	https://raw.githubusercontent.com/animesh/scripts/bdabc21a58bae5b1fe4eb3e6ada01a677eda2e20/proteinGroupsQC.r	https://raw.githubusercontent.com/animesh/scripts/7bd19ef074bfd97d04c55741d519be6460534558/evidenceQC.r
Murine PDAC spheroids	TIMSTOF/LARS/2022/APRIL/Mathilde/220427_Mathilde_15_Slot2-1_1_1585.d	PXD057804	https://raw.githubusercontent.com/animesh/scripts/6422947cbc4759f52ba821812203edd687941e85/proteinGroupsQC.r	https://raw.githubusercontent.com/animesh/scripts/cb031f827be437a5c3b6b60fbdc0ad55e5bd00eb/evidenceQC.r
Murine PDAC spheroids	TIMSTOF/LARS/2022/APRIL/Mathilde/220427_Mathilde_30_Slot2-2_1_1586.d	PXD057804	https://raw.githubusercontent.com/animesh/scripts/6422947cbc4759f52ba821812203edd687941e85/proteinGroupsQC.r	https://raw.githubusercontent.com/animesh/scripts/cb031f827be437a5c3b6b60fbdc0ad55e5bd00eb/evidenceQC.r
Murine PDAC spheroids	TIMSTOF/LARS/2022/APRIL/Mathilde/220427_Mathilde_60_Slot2-3_1_1587.d	PXD057804	https://raw.githubusercontent.com/animesh/scripts/6422947cbc4759f52ba821812203edd687941e85/proteinGroupsQC.r	https://raw.githubusercontent.com/animesh/scripts/cb031f827be437a5c3b6b60fbdc0ad55e5bd00eb/evidenceQC.r
Human PDAC organoids	TIMSTOF/LARS/2023/230815 mathilde/230815_mathilde_1_Slot2-10_1_4885.d	PXD057928	https://raw.githubusercontent.com/animesh/scripts/bc67363110aec2ac64ca2ac5498f35e8a8c89900/proteinGroupsQC.r	https://raw.githubusercontent.com/animesh/scripts/a28de9f76919272c6805cfd63bd4c5cf7159416f/evidenceQC.r
Human PDAC organoids	TIMSTOF/LARS/2023/230815 mathilde/230815_mathilde_2_Slot2-11_1_4887.d	PXD057928	https://raw.githubusercontent.com/animesh/scripts/bc67363110aec2ac64ca2ac5498f35e8a8c89900/proteinGroupsQC.r	https://raw.githubusercontent.com/animesh/scripts/a28de9f76919272c6805cfd63bd4c5cf7159416f/evidenceQC.r

Human PDAC organoids	TIMSTOF/LARS/2023/230815 mathilde/230815_mathilde_3_Slot2-12_1_4889.d	PXD057928	https://raw.githubusercontent.com/animesh/scripts/bc67363110aec2ac64ca2ac5498f35e8a8c89900/proteinGroupsQC.r	https://raw.githubusercontent.com/animesh/scripts/a28de9f76919272c6805cfd63bd4c5cf7159416f/evidenceQC.r
Human PDAC tumor	TIMSTOF/LARS/2024/240404_Mathilde/240404_Mathilde_TN_PDAC_P1_Slot2-4_1_7011.d	PXD057607	https://raw.githubusercontent.com/animesh/scripts/991aa9f5edeccf382973c32a81f9ec92a80cc350/proteinGroupsQC.r	https://raw.githubusercontent.com/animesh/scripts/4fb1bb0ca0817151e72ddfc9f15585c1f1011bf3/evidenceQC.r
Human PDAC tumor	TIMSTOF/LARS/2024/240404_Mathilde/240404_Mathilde_TN_PDAC_P2_Slot2-5_1_7013.d	PXD057607	https://raw.githubusercontent.com/animesh/scripts/991aa9f5edeccf382973c32a81f9ec92a80cc350/proteinGroupsQC.r	https://raw.githubusercontent.com/animesh/scripts/4fb1bb0ca0817151e72ddfc9f15585c1f1011bf3/evidenceQC.r
Human PDAC tumor	TIMSTOF/LARS/2024/240404_Mathilde/240404_Mathilde_TN_PDAC_P4_Slot2-7_1_7017.d	PXD057607	https://raw.githubusercontent.com/animesh/scripts/991aa9f5edeccf382973c32a81f9ec92a80cc350/proteinGroupsQC.r	https://raw.githubusercontent.com/animesh/scripts/4fb1bb0ca0817151e72ddfc9f15585c1f1011bf3/evidenceQC.r
Murine pancreatic exocrine organoids	TIMSTOF/LARS/2023/230414 mathilde/230414_Mathilde_1_Slot2-46_1_4326.d	PXD057829	https://raw.githubusercontent.com/animesh/scripts/8d72cbdf70faaab63e7e594eaa81dfdd9a1487cf/proteinGroupsQC.r	https://raw.githubusercontent.com/animesh/scripts/bec0b9b9a0c03317bd2c605938b83163f8ea45a2/evidenceQC.r
Murine pancreatic exocrine organoids	TIMSTOF/LARS/2023/230414 mathilde/230414_Mathilde_2_Slot2-47_1_4328.d	PXD057829	https://raw.githubusercontent.com/animesh/scripts/8d72cbdf70faaab63e7e594eaa81dfdd9a1487cf/proteinGroupsQC.r	https://raw.githubusercontent.com/animesh/scripts/bec0b9b9a0c03317bd2c605938b83163f8ea45a2/evidenceQC.r
Murine PDAC organoids	'TIMSTOF/LARS/2023/230414 mathilde/230414_Mathilde_3_Slot2-48_1_4330.d	PXD057888	https://raw.githubusercontent.com/animesh/scripts/476604c6f638dd6276ee1a24837d6c5bea212503/proteinGroupsQC.r	https://raw.githubusercontent.com/animesh/scripts/476604c6f638dd6276ee1a24837d6c5bea212503/evidenceQC.r

2. **Murine PDAC cell culture procedure**

The UN-KC-6141 cell line originates from a pancreatic tumor in a genetically modified mouse designed to mimic human pancreatic cancer. This mouse, carrying the KrasG12D; Pdx1-Cre mutation, was 50 weeks old when the tumor was harvested. This process ensures the cell line retains crucial genetic features and characteristics for research on pancreatic cancer.

Additionally, it also maintains the epithelial characteristics and drug resistance patterns observed in human pancreatic ductal adenocarcinoma, thereby offering a valuable model for preclinical studies and therapeutic testing [1]. UN-KC-6141 is an adherent cell line and have a doubling time of 70 hours[1]. The required reagents, materials and equipment for handling the UN-KC-6141 are described in table S1.

Subculture and thawing:

1. Thaw UN-KC-6141 cells from liquid nitrogen in 37 °C water bath for 1-2 minutes by swirling. The cryotube are cryopreserved with ~2.0 x 10^6 cells. The cells are frozen in growth media (80%), with 10% FBS and 10% DMSO.
2. Transfer cells from cryotube to 4 mL pre-heated growth media (Table S1) in a 15 mL centrifuge tube.
3. Centrifuge cells at 200 x g for 5 min at RT.
4. Discard the supernatant and add 5 mL fresh pre-heated growth media (Table S1) to the cells. Resuspend the cells thoroughly to obtain single cell suspension (NB: cells are "clustering", and they can handle some resuspensions).
5. Transfer to a T_{25} flask and incubate overnight.
6. Split the culture the next day and transfer to T_{75} flask using a low split number (e.g. 1:2 or 1:3). Keep culture incubated at a humidified incubator holding 37°C and 5% CO_2.

Passage:

1. UN-KC-6141 cells are better to be split at ~70-80 % confluency due to rapid growth.
2. Remove and discard medium, and wash with 5 mL pre-heated PBS and detach cells using 2 mL pre-heated Trypsin-EDTA solution.
3. Incubate at 37°C for 5 minutes, then examine under a microscope to verify detachment (cells at lower passage numbers detach more readily). If necessary, apply mechanical force by gently tapping the flask to release the cells from the surface. Stop trypsinization with 4 mL growth medium (trypsin 1:2 medium).
4. Transfer to 15 mL tube and centrifuge at 200 x g for 5 min at RT.
5. Discard the supernatant and add growth medium. As soon as the culture is established, UN-KC-6141 cells are easily split every 2-3 days (Fig. S1) at a split number of 1:10 or higher.

Table S1. Materials and reagents for cultivation of UN-KC-6141 cells

Equipment				
Cell culture flasks, T25 cm2 and T75 cm2				
Incubator at 37 °C with 5% CO2				
Serological pipettes (5 mL, 10 mL, 20 mL)				
Sterile pipette tips				
70% Ethanol				
Cultivation flasks (Corning® 75cm^2 U-Shaped Canted Neck Cell Culture Flask with Plug Seal Cap)				
**Medium**	**Reagents**	**Manufacturer**	**Cat number**	**Volume**

Growth media	Dulbecco's Modified Eagle's Medium - high glucose 4.5 g/L	Sigma Aldrich	D6429-500ML	89 mL
	Fetal Bovine Serum, 10%	Sigma Aldrich	F7524	10 mL
	Penicillin-Streptomycin, 1%	Sigma Aldrich	P4333	1 mL
For cultivation and freezing	Trypsin-EDTA	Life technologies limited	25300-054	
	Dimethyl sulfoxide (DMSO)	Calbiochem®	317275	
	Dulbecco's Phosphate Buffered Saline (DPBS)	Life technologies	D8537	

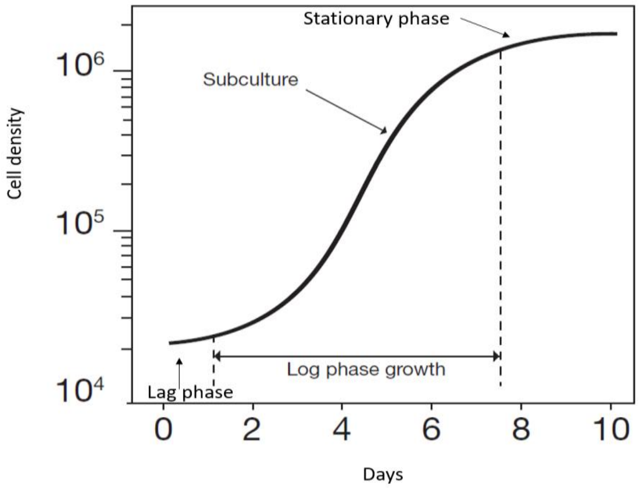

Fig. S1 Growth pattern of cultured cell. Semilogarithmic plot showing cell density versus time (days) in culture. Cells typically follow this growth pattern: starting with a lag phase of slow growth, then entering an exponential log phase after adapting to the environment. When space or nutrients become limited, growth ceases. Optimal timing for subculturing/passaging is indicated (Thermo Fisher Cell Culture Basics Handbook). Figure edited by the author in BioRender.

3. Murine PDAC spheroid formation procedure

Mouse PDAC spheroids were generated using the UN-KC-6141 cells described above, employing the same reagents and cultivation methods as described therein. The spheroids were formed by using the hanging drop method, and passage 39 was used for this purpose. The drops were placed into the underside of the lid of a petri dish, and subsequently inverted so that each drop was held in place by surface tension and gravity. The droplets consisted of 60 000cells/27µl and the dishes were filled with 1 ml medium, to avoid evaporation and provide nutrition. All samples were cultured and kept in a humidified incubator of 37°C and 5% CO2. The spheroids were collected for proteomic analysis after 7 days.

1. Follow the protocol for passaging described in “PDAC cell culture”. Following step 4, introduce a precise volume of full growth media to attain a cell density of 60,000 cells per 27µl for the placement of droplets.
2. Add 1 mL of full growth media to the dish.
3. Place the droplets on the underside of the lid (6 droplets per lid).
4. Carefully invert the lid to cover the dish with full growth media.
5. Observe the formation of spheroid growth under an inverted microscope and change the media in the petri dish to avoid evaporation every other day. To prevent disruption of the spheroids during harvesting for proteomics, utilize a pipette tip with the end cut off (Fig. S2). In total 15 droplets were collected for proteomic analysis.

Spheroids follow a growth pattern similar to solid tumors, where the growth stagnates as spheroids reach a diameter of 500-1000 µm, known as the spheroid growth plateau (Fig. S2). At this point, solid tumors typically begin vascular growth in response to factors like VEGF and extracellular matrix construction[2].

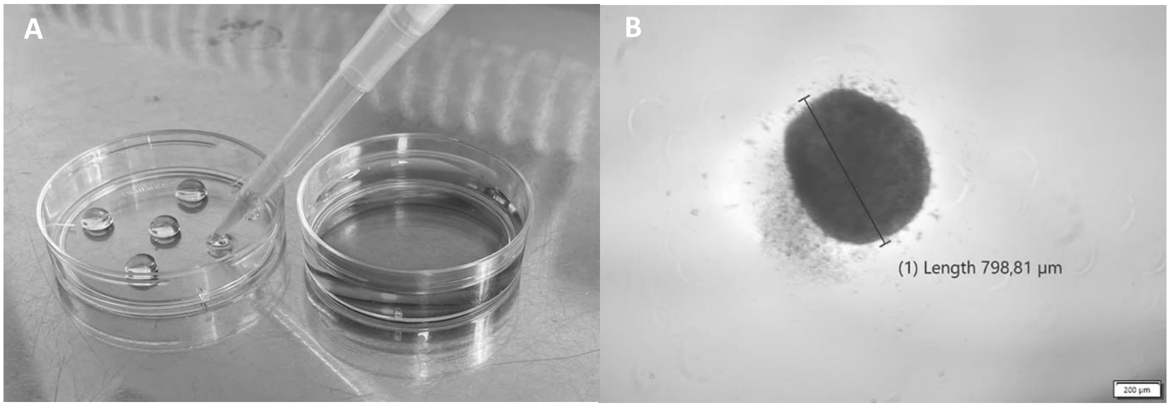

Fig. S2. Sample collection of spheroids and morphology. Spheroids in culture medium prior to proteomic analysis, being handled with a pipette tip cut to avoid disruption (A). Microscopic image of a spheroid at day 3 with a measured diameter of approximately 798,81 µm captured with Olympus CKX53 iPC microscope (B).

4. Murine organoid cultivation procedure

The murine organoids are cultivated in a similar manner, with slightly different splitting medium (Table S2-S4).

Murine pancreatic exocrine organoids: The pancreatic exocrine organoids used in this study were purchased from Stem Cell Technologies. Every vial holds enough fragments of 200 mouse pancreatic exocrine organoids to initiate cultures in two wells. These organoids originate from the pancreas of C57BL/6 mice (Catalog #70933). Organoids are ready for passage when the majority reach a size between 100 µm and 200 µm and their lumens start to darken, signaling optimal growth and the need for subculturing.

Murine PDAC organoids: The mouse PDAC organoids utilized in this study were generously provided by Cold Spring Harbor Laboratory. They were created by isolating pancreatic ductal adenocarcinoma tissue from C57BL/6 mice according to the Tuveson Laboratory Murine and Human Organoid Protocols (http://tuvesonlab.labsites.cshl.edu/wp-content/uploads/sites/49/2018/06/20170523_OrganoidProtocols.pdf).

Mouse pancreatic organoids grow faster than human organoids, and the cancerous mouse organoids exhibit even more rapid growth, necessitating a higher splitting ratio during passaging. Typical split ratios for mouse PDAC organoids range from 1:4 to 1:8, based on the confluency of the organoids [3].

Preparation of media

Add Y-27632 (ROCK inhibitor) upon thawing and during any stress-prone stages to enhance organoid viability by stabilizing the cytoskeleton and maintaining cellular interactions. The ROCK inhibitor is crucial in the thawing process of organoids because it helps to mitigate the stress and prevent apoptosis induced by the freezing and thawing cycle, thus enhancing cell survival and improving overall recovery. This inhibitor plays a key role in stabilizing the cytoskeleton and maintaining cell-cell and cell-matrix interactions, which are essential for the structural integrity and functionality of the organoids. A working solution of 10mM ROCK inhibitor is added to 1-2 first passages after thawing and splitting.

Preparation of media

Prepare the media before initiating the culturing process.

1. For preparation of the complete growth media, thaw the supplement at 2-8°C overnight and mix well before use. The PancreaCult OGM Mouse Supplement was aliquoted and stored at -20°C is not used right away according to StemCells description.
2. Combine 5 mL of the thawed supplement with 95 mL of Basal Medium.
3. Add antibiotics (50 µg/mL gentamicin) and ensure thorough mixing. Allow the mixture to reach room temperature (5-25°C) before application.
4. Prepare splitting media by mixing 2 mL 25% BSA (dissolved in sterile water) with 48 mL DMEM/F-12 with 15mM HEPES.

Cultivation

Pre-warm a 24-well tissue culture plate by placing it in a 37°C incubator for a minimum of one hour. Simultaneously, chill a box of sterile 200µl pipette tips by storing it at 2-8°C and thaw Matrigel on ice. The pipette tips are pre-cooled with cold PBS or Anti adherence. Aliquots of 200µl Matrigel takes at least one hour to thaw.

1. Prewarm complete growth media to room temperature (15-20°C).
2. Thaw organoids using a thawing box: Heat 150 mL of sterile water in a water bath and then place it in an incubator set to 37°C and 5% CO_2. Move the organoids from liquid nitrogen storage to the pre-warmed water and allow them to thaw in the incubator for 2-3 minutes. Clean the exterior of the vial with 70% alcohol.
3. Add 1 mL splitting medium (RT) to the cryovial and gently resuspend by pipetting 4-6 times, and ensure the vial is thoroughly rinsed.
4. Centrifuge the organoids at 300 x g for 5 minutes for 5 minutes at 4°C.
5. Discard the supernatant and resuspend the pellet in 62 µL Matrigel using pre-cooled pipette tips.
6. Seed 32 µL Matrigel domes onto a pre-heated 24-well plate.
7. Incubate the culture plate at 37°C, 5% CO_2 for 12 min. Invert the plate for the last 2-3 min of the incubation time to maintain the 3D shape of the domes.
8. Carefully add 550 µl complete growth media against the side of the well to avoid disrupting the domes and place the culture plate back into the incubator.
9. Change medium every 2-3 days. Organoids are ready for passage after 4-7 days.

Passage: During the splitting process, ensure that all reagents and culture plates are kept on ice. The quantity of cells or domes required varies based on the specific experimental design and the splitting interval. Typically, we use a range of 10,000 to 30,000 cells per dome is used. To enhance the dissolution of Matrigel, the splitting medium can be substituted with cell recovery solution.

10. Pre-warm a 24-well tissue culture plate by placing it in a 37°C incubator for a minimum of one hour. Simultaneously, chill a box of sterile 200µl pipette tips by storing it at 2-8°C and thaw Matrigel on ice. Aliquots of 200µl Matrigel takes at least one hour to thaw.
11. Discard medium from the wells without disrupting the domes.
12. Add 500 µL cell recovery solution into each well. Disrupt the Matrigel domes with a sterile pipette and break the Matrigel domes using a sterile pipette tip and mix thoroughly.
13. Transfer the solutions to a 15 mL tube.
14. Rinse the wells using 500 µL of cell recovery solution. This step can be repeated if necessary.
15. Centrifuge the suspension at 500 x g for 8 minutes at 4°C.
16. Gently resuspend the pellet in 5 mL of splitting medium brought to room temperature, and then transfer it to a 50 mL tube.
17. Position a needle-tip against the inner wall of the 50 mL tube and carefully push the suspension through it.
18. Transfer 10 µL of the suspension into a microtube and combine it with 10 µL of Trypan Blue. Then, place 10 µL of this mixture onto a counting slide for cell counting.
19. Transfer the cell suspension to a 15 mL tube and centrifuge at 500 x g for 8 min at 4°C, and thereafter discard the supernatant.
20. Based on the cell counting, adjust the volume of Matrigel to be added in order to attain a density of 10,000 to 30,000 cells per dome
21. Seed Matrigel domes (32 µL) onto a pre-heated 24-well plate.
22. Place the culture plate in an incubator at 37°C and 5% CO_2 for 12 minutes. For the final 2-3 minutes of incubation, invert the plate to help preserve the three-dimensional shape of the domes.
23. Change media every 2-3 days by aspirating the medium and adding 550 µl fresh complete growth media. Split after 4-7 days.

Table S2. Equipment and material for working with organoids

Equipment and Material	Manufacturer	Cat number
Centrifuge	Hettich Rotina 420R Centrifuge	
	Eppendorf 5415R Refrigerated Centrifuge VWR international	
	Kubota 5420 230V	
Sterile pipette tips with filter (0,5-1000 µL)	Biotix	
Cell counter (Countess™ automated cell counter)	Invitrogen	
EVE™ Cell counting slide	NanoEntek	EVS-050
Syringe (15-50 mL)	Braun (Omnifix®)	4616200V

BD™ Blunt Fill Needle	Becton Dickinson S.A.	303129
Sterile Filter VWR®, Bottle-Top Vaccum Filtration Systems, PES, 0.2 µm Pore size	VWR	514-0332
Costar 24-well Flat-Bottom Plate, Tissue Culture-Treated	Costar	38017
Falcon Conical Tubes, 15 mL, 50 mL		
Anti Adherence Rinsing Solution	StemCell	07010
Dulbecco's Phosphate Buffered Saline (DPBS)	Life technologies	D8537
Corning Matrigel Growth Factor Reduced (GFR) Basement Membrane Matrix, Phenol Red-Free LDEV-Free	Corning	356231
Cell Recovery Solution	Corning	354253
Y-27632	StemCell Technologies	72302
Mr. Frosty Freezing Container	Thermo Fisher	5100-0001
TrypLE™ Select	Gibco™	12563011
CryoTube™	Thermo Fischer Scientific	366656

Table S3. Materials and reagents for cultivation of the normal mouse organoids

Medium	Reagents	Manufacturer	Cat number	volume
Mouse Pancreatic Organoids		StemCell Technologies	70933	
Splitting media	Dulbecco's Modified Eagle's Medium/Nutrient Ham's Mixture F-12 (DMEM/F-12) with 15 mM HEPES buffer	StemCell Technologies	36254	48 mL
	Sterile 25% Bovine serum albumin (BSA)	Sigma Aldrich	Cas 9048-46-8	2 mL
Complete growth media	Basal medium	StemCell Technologies	06041	95 mL
	PancreaCult OGM Mouse Supplement	StemCell Technologies	06040	5 mL

	Gentamicin	Sigma Aldrich	Cas 1405-41-0	1 mL

Table S4. Materials and reagents for cultivation of murine PDAC organoids.

Medium	Reagents	Manufacturer	Cat number	volume
Cold Spring Harbor Laboratory				
Splitting media	Advanced DMEM F:12	Thermo Fischer Scientific	12634028	485 mL
	HEPES (10 mM)	Thermo Fischer Scientific	15630106	5 mL
	Penicillin-Streptomycin (100 U/ mL)	Thermo Fischer Scientific	11548876	5 mL
	GlutaMAX	Thermo Fischer Scientific	35050061	5 mL
	Sterile 25% Bovine serum albumin (BSA)	Sigma Aldrich	Cas 9048-46-8	20 mL
Complete growth media	Basal medium	StemCell Technologies	06041	95 mL
	PancreaCult OGM Mouse Supplement	StemCell Technologies	06040	5 mL
	Gentamicin	Sigma Aldrich	Cas 1405-41-0	1 mL

5. Human PDAC organoid cultivation procedure

Mouse and human PDAC organoids necessitate specialized media for cultivation, splitting and freezing. Specifically, the cultivation of human PDAC organoids demands the inclusion of Wnt-3a and Rspondin1 conditioned media, which in turn requires the prior cultivation of Wnt-3a and R-spondin-producing cells. The cultivation takes 2-3 weeks and must be undertaken well in advance of the organoid cultivation process

The organoids depicted (Model: HCM-CSHL-0094-C25, ATCC No. PDM-41) were acquired from ATCC, derived from the primary pancreatic tissue obtained from the pancreatic head of a 61-year-old female. The patient, whose tissue was used to generate these organoids, was diagnosed with Stage III pancreatic ductal adenocarcinoma, classified histologically as adenocarcinoma ductal type. Notably, the patient had not undergone any neoadjuvant therapy at the time of organoid derivation. This model offers an advanced stage representation of the disease, crucial for studying the pathophysiology and therapeutic responses in a clinically relevant context.

Preparation of Wnt-3a conditioned medium: The Wnt-3A cell line (ATCC® CRL-2647™) originates from the areolar adipose tissue of a 100-day-old male mouse, demonstrating fibroblast-like morphology. It is developed and optimized specifically to produce Wnt-3A

conditioned medium, according to the methodology described by R. Nusse[4] . This cell line is maintained under strict laboratory conditions, emphasizing its use exclusively for research purposes[4]. The protocol for production of Wnt-3A conditioned media was obtained from the vendor and the Tuveson Laboratory Murine, Human Organoid Protocols (http://tuvesonlab.labsites.cshl.edu/wp-content/uploads/sites/49/2018/06/20170523_OrganoidProtocols.pdf) and the laboratory protocol previously described[5]. To assure the quality of the conditioned media HEK293 cells were cultured and passaged in medium supplemented with geneticin, then seeded into 96-well plates for a TCF/LEF reporter assay to evaluate Wnt/R-spondin pathway activation. After treatment with various concentrations of Wnt and R-spondin conditioned media, cells underwent a luciferase assay to measure pathway activation, reflecting the degree of cellular response.

Preparation of the Wnt-3a conditional media and the timeline of the Wnt-3a culturing process are illustrated (Table S5 and Fig. S3). This culturing protocol is tailored for initiating the process by thawing one ampulla containing 1.5 million cells.

Table S5. Materials and reagents for production of Wnt-3a conditioned media

Medium	Reagents	Manufacturer	Cat number	Volume
L Wnt-3A		ATCC	CRL-2647™	
Growth medium	Dulbecco`s Modified Eagle`s Medium (DMEM), high glucose, GlutaMAX supplement, pyruvate	Thermo Fischer Scientific	31966	90 mL
	Fetal calf serum (FCS), 10%			10 mL
Complete growth medium	Dulbecco`s Modified Eagle`s Medium (DMEM), high glucose, GlutaMAX supplement, pyruvate	Thermo Fischer Scientific	31966	446 mL
	Fetal calf serum (FCS), 10%			50
	Geneticin selective Antibiotic. C(G418 Sulfate) (0.8%)	Thermo Fischer Scientific	11548876	4 mL
Wnt-3A conditioned medium	CM1 and CM2 are mixed 1:1			915,33 mL
	Bovine serum albumin (BSA), 1%	Sigma Aldrich	Cas 9048-46-8	9,64 mL
	GlutaMAX, 1%	Thermo Fischer Scientific	35050061	9,64 mL
	HEPES (10 mM), 1%	Thermo Fischer Scientific	15630106	9,64 mL

	Penicillin-Streptomycin (100 U/ mL), 1%	Thermo Fischer Scientific	11548876	9,64 mL
	N-2 supplement, 1%	Thermo Fischer Scientific	17502001	9,64 mL
	B27 supplement, 2%)	Thermo Fischer Scientific	17504001	19,28 ml

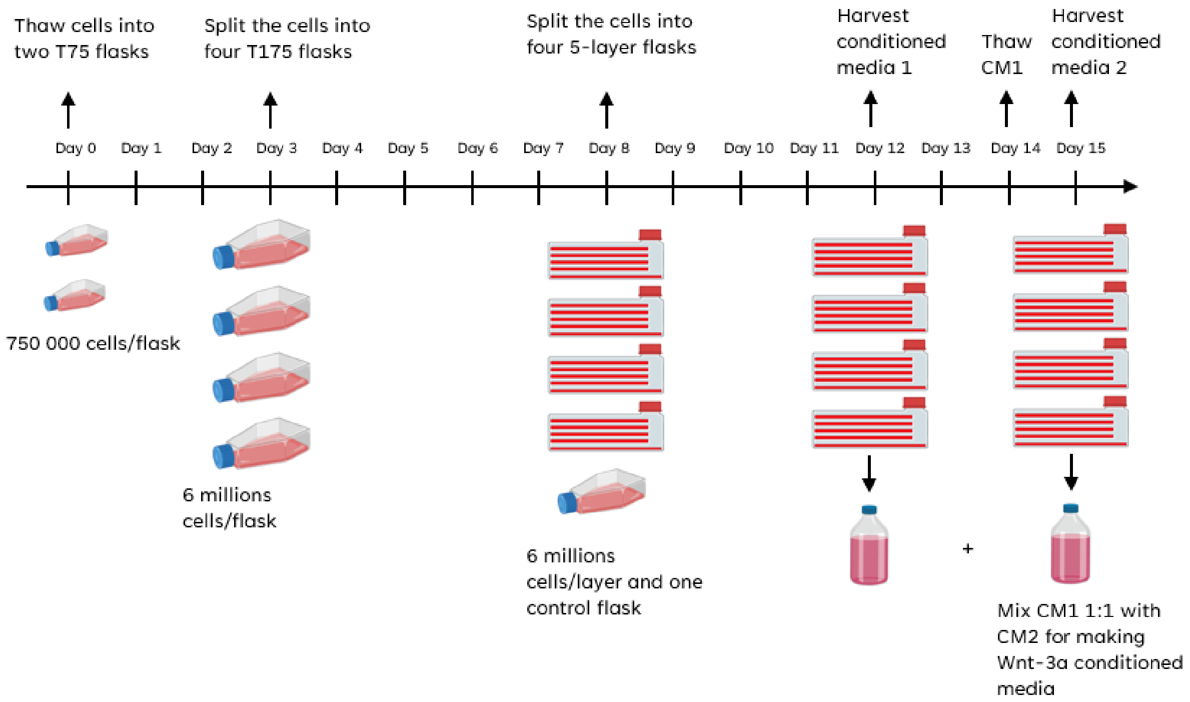

Fig. S3 The cultivation process for Wnt-3A cells. This timeline is tailored for thawing for 1.5 million cells. 5-layer flasks were utilized to produce greater volumes and simplify the ease of use.

1. Thaw cells using a thawing box: Heat 150 mL of sterile water in a water bath and then place it in an incubator set to 37°C and 5% CO2. Move the cells from liquid nitrogen storage to the pre-warmed water and allow them to thaw in the incubator for 2-3 minutes. Clean the exterior of the vial with 70% alcohol.
2. Add 1 mL splitting medium pre-adjusted to room temperature to the cryovial and gently resuspend by pipetting 4-6 times, and ensure the vial is thoroughly rinsed.
3. Transfer the cells into a 15 mL tube filled with 8 mL of pre-warmed complete growth medium. Use an additional 1 mL of the same medium to rinse the original vial, ensuring all cells are collected.
4. Centrifuge the suspension at 400 x g in (RT) for 5 min.
5. Discard the supernatant and add 2 mL complete growth medium.
6. Add 11 mL pre-heated complete growth medium to two T75 culture flasks and transfer 1 mL of the suspension to each flask. Place the culture flasks in the incubator at 37°C with 5% CO_2 and monitor the growth of the cells under a microscope until confluent.

7. On day 3 (when confluency is reached), heat 100 mL complete growth media in a water batch at 37°C.
8. Discard the medium from the two T75 flasks and wash with 4 mL DPBS.
9. Add 3 mL trypsin to each flask and place the cells in an incubator holding 37°C and 5% CO_2 for 5-10 minutes. Add 4 mL complete growth medium to each flask to neutralize the trypsin.
10. Transfer the suspension from each flask to a 15 mL tube and centrifuge the tube at 400 x g in room temperature for 8 min.
11. Discard the supernatant and add 4,34 mL pre-heated complete growth medium to each tube (for split ratio 1:10). Count the cells (seed ~6 millions/flask for maintaining the timeline described in Figure X).
12. Add 22.5 mL pre-heated complete growth medium to four T175 flasks and transfer 1 mL cell suspension to each T175 flask.
13. Incubate the culture flasks in an incubator holding 37°C and 5% CO_2 until confluency is reached. Typically, day 8 for cell numbers described in this protocol.
14. On day 8, add 112,5 mL growth medium to each of the four 5-layer flask and place in incubator to allow the medium to pre-heat.
15. Discard the medium from the four T175 flasks and wash with 4 mL DPBS.
16. Add 6 mL trypsin to each flask and place the cells in an incubator holding 37°C and 5% CO_2 for 5-10 minutes. Add 12 mL growth medium to each flask to neutralize the trypsin.
17. Centrifuge the cell suspension from each flask in a 50 mL tube at 400 x g for 8 min at RT.
18. Discard the supernatant and resuspend in 10 mL growth medium. Count the cells (seed ~6 millions/layer for maintaining the timeline described in Figure S3).
19. Transfer 5 mL of the cell suspension to each of the four 5-layer flasks. The 5-layer flasks will now contain 117,5 mL. Remember to include one T175 control flask to monitor the cell confluency.
20. On day 12, when confluency is reached the conditioned media 1 (CM1) can be harvested.
21. Transfer the growth medium from the 5-layer flasks and control flask into 50 mL tubes and centrifuge tubes at 400 x g for 8 min at RT. Aspirate supernatant from all the 50 mL tubes to a sterile 500 mL glass bottle and freeze at -20°C, and mark as CM1.
22. Add 117.5 mL fresh preheated growth medium back to the 5-layer flasks containing the cells.
23. Incubate the flasks at 37°C, 5% CO_2 for 3-4 days.
24. One day 14 place the bottle containing CM1 at 4°C to defrost overnight (24 hours) before harvesting conditioned medium 2 (CM2).
25. On day 15 harvest CM2 from the five-layer flasks in the same manner as CM1 was previously harvested. Then, transfer the harvested CM2 into a glass bottle large enough to accommodate both batches and mix them in a ratio 1:1.
26. Add factors as described in Table 5 to prepare Wnt-3A conditioned medium.
27. Filter the Wnt-3A conditioned medium through a sterile 0.22 µm filter, and freeze in working volumes at -20°C.

Preparation of Rspondin-1 conditioned medium: This culturing protocol was tailored for initiating the process by thawing one ampulla containing 10 million Rspondin1 cells (Table S6 and Fig. S4).

Table S6. Materials and reagents for production of Rspondin1 conditioned media

Medium	Reagents	Manufacturer	Cat number	Volume

293T-HA-rspo-Fc (P5)		AMS Biotechnology, Abington, United Kingdom	AMS. RSPO1-CELLS (RRID: CVCL_RU08)	
Growth medium	Dulbecco`s Modified Eagle`s Medium (DMEM), high glucose, GlutaMAX supplement, pyruvate	Thermo Fischer Scientific	31966	90 mL
	Fetal calf serum (FCS), 10%			10 mL
	Gibco Penicillin-Streptomycin (10 000 U/ mL)	Thermo Fischer Scientific	11548876	
Complete growth medium	Dulbecco`s Modified Eagle`s Medium (DMEM), high glucose, GlutaMAX supplement, pyruvate	Thermo Fischer Scientific	31966	443,5 mL
	Fetal calf serum (FCS), 10%			50 mL
	Gibco Penicillin-Streptomycin	Thermo Fischer Scientific	11548876	5 mL
	Zeocin (100mg/ml stock)	Invitrogen	R25001	1,5 mL
AD-DF++ medium	Advanced DMEM F:12	Thermo Fischer Scientific	12634028	698,5 mL
	HEPES (10 mM), 1%	Thermo Fischer Scientific	15630106	6,99 mL
	GlutaMAX supplement (100X), 1%	Thermo Fischer Scientific	35050061	6,99 mL
	Gibco Penicillin-Streptomycin	Thermo Fischer Scientific	11548876	6,99 mL
RSPO1 conditioned medium	AD-DF++ medium is harvested from the culture flasks			649,58 mL
	Bovine serum albumin (BSA), 1%	Sigma Aldrich	Cas 9048-46-8	6,99 mL
	GlutaMAX, 1%	Thermo Fischer Scientific	35050061	6,99 mL
	HEPES (10 mM), 1%	Thermo Fischer Scientific	15630106	6,99 mL
	Penicillin-Streptomycin 1%	Thermo Fischer Scientific	11548876	6,99 mL
	N-2 supplement (1X, 1%)	Thermo Fischer Scientific	17502001	6,99 mL

	B27 supplement (2X, 2%)	Thermo Fischer Scientific	17504001	13,97 mL

1. Thaw cells using a thawing box: Heat 150 mL of sterile water in a water bath and then place it in an incubator set to 37°C and 5% CO2. Move the cells from liquid nitrogen storage to the pre-warmed water and allow them to thaw in the incubator for 2-3 minutes. Clean the exterior of the vial with 70% alcohol.

2. Add 1 mL room splitting medium pre-adjusted to room temperature to the cryovial and gently resuspend by pipetting 4-6 times, and ensure the vial is thoroughly rinsed.

3. Transfer the cells into a 15 mL tube filled with 8 mL of pre-warmed complete growth medium. Use an additional 1 mL of the same medium to rinse the original vial, ensuring all cells are collected.

4. Centrifuge the suspension at 400 x g in room temperature (RT) for 5 min.
5. Discard the supernatant and add 2 mL complete growth medium.
6. Add 22,5 mL pre-heated complete growth medium to two T175 culture flasks and transfer 1 mL of the suspension to each flask. Place the culture flasks in the incubator at 37°C with 5% CO_2 and monitor the growth of the cells under a microscope until confluency.
7. On day 6, add 112,5 mL growth medium to three 5-layer flasks and place in incubator to allow the medium to pre-heat.
8. Heat 45 mL complete growth media in a water batch at 37°C.
9. Discard the medium from the two T175 flasks and wash with 4 mL DPBS.
10. Add 6 mL trypsin to each flask and place the cells in an incubator holding 37°C and 5% CO_2 for 5-10 minutes. Add 12 mL complete growth medium to each flask to neutralize the trypsin.
11. Centrifuge the cell suspension from each flask in a 50 mL tube at 400 x g for 8 min at RT.
12. Discard the supernatant and resuspend in 10 mL growth medium. Count the cells (seed ~6 millions/layer for maintaining the timeline described in Figure X).
13. Transfer 5 mL of the cell suspension to each of the three 5-layer flasks. The 5-layer flasks will now contain 117,5 mL. Remember to include one T175 control flask to monitor the cell confluency.
14. On day 11, when confluency is reached, discard the medium from the 5-layer flasks.
15. Add AD-DF++ medium (50mL/layer), 225 ml in total for one 5-layer flask.
16. Place the culture flasks in an incubator holding 37°C and 5% CO_2 for one week.
17. Harvest the AD-DF++ medium from the 5-layer flasks and transfer the suspension from each flask to 50 mL tubes and centrifuge the tubes at 400 x g in room temperature for 8 min.
18. Aspirate supernatant from all the 50 mL tubes to a sterile 500 mL glass bottle and add factors as described in Table 6 to prepare Rspo1 conditioned medium.
28. Filter the Rspo1 conditioned medium through a sterile 0.22 µm filter, and freeze in working volumes at -20°C.

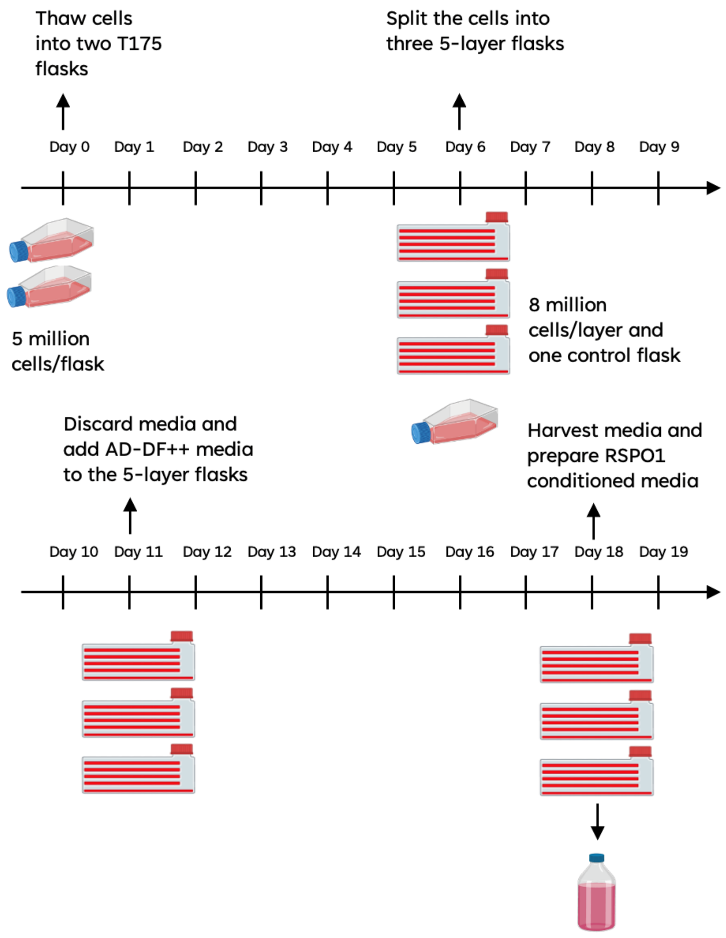

Fig. S4 The cultivation process for RSPO1 cells. This timeline is tailored for initiating the process by thawing one ampulla containing 10 million Rspondin1 cells into two T175 flasks.

Human organoids proliferate more slowly compared to mouse organoids, and thrive better at higher densities, the recommended splitting ratios should generally vary between 1:1 and 1:3 to maintain optimal culture health[3].

Note that human PDAC organoids necessitate specialized media formulations to sustain their growth effectively, described in Table S7.

Human PDAC organoids are cultivated in the same manner as the murine organoid cultivation procedure. However, if experiencing trouble achieving single cells, an additional trypzination step might be included. This is particularly important if doing experiments requiring single cells. This step can be included after removal of the Matrigel suspension, by adding 2 mL TrypLE to the pellet. Heat the tube containing the cell suspension in a water bath at 37°C for 5 minutes and thereafter add 4 mL splitting medium to neutralize the TrypLE. Centrifuge the suspension at 500 x g for 8 minutes at 4°C and discard the supernatant. Continue by adding 3 mL fresh room tempered splitting medium and transfer to a 50 mL tube for using the needle and follow the protocol as previously described in the murine organoid cultivation procedure. Similarly to the mouse organoids, ROCK inhibitor should be added upon thawing and during any stress-prone

stages to enhance organoid viability by stabilizing the cytoskeleton and maintaining cellular interactions.

Table S7. Materials and reagents for production of human PDAC organoid media.

Name	Reagents	Manufacturer	Cat number	Final concentration	Volume
Human PDAC organoids PDM-41		ATCC	HCM-CSHL-0094-C2<br>Lot 70014508		
Splitting media	Advanced DMEM F:12	Thermo Fischer Scientific	12634028		500 mL
	HEPES pH 7.2-7.5	Thermo Fischer Scientific	15630106		5 mL
	GlutaMAX Supplement	Thermo Fischer Scientific	35050061		5 mL
	Primocin 50 mg/mL	InvivoGen	ant-pm-2		1 mL
	Bovine serum albumin (BSA)	Sigma Aldrich	Cas 9048-46-8		1,67 mL
Supplemented basal media	Advanced DMEM:F12	Thermo Fischer Scientific	12634028		90 mL
	HEPES (10 mM)	Thermo Fischer Scientific	15630106		2,5 mL
	L-Glutamine Solution, 200 mM	ATCC	30-2214		2,5 mL
	B27 supplement	Thermo Fischer Scientific	17504001		5 mL
Complete growth medium	* RSPO1 Conditioned Media			10 %	10 mL
	**Wnt-3A conditioned Media			50 %	50 mL
	Supplemented basal media			37,4 %	37,4 mL
	***Organoid growth kit 1B	ATCC	ACS-7101		
	Noggin	ATCC	ACS-7200	100ng/mL	0,2 mL
	EGF	ATCC	ACS-7202	50 ng/mL	0,1 mL

		Nicotinamide	ATCC	ACS-7214	10 mM	1 mL
		N-Acetyl-cysteine	ATCC	ACS-7215	1.25 mM	0,25 mL
		FGF-10	ATCC	ACS-7204	100 ng/mL	1 mL
		Gastrin	ATCC	ACS-7208	10 nM	0,02 mL
		A 83-01	ATCC	ACS-7209	500 nM	0,02 mL
Freezing media	Dimethyl sulfoxide (DMSO)		Calbiochem®	317275	5%	20 mL
	Fetal calf serum (FCS)				95%	1 mL

* See Table S5 Materials and reagents for production of Wnt-3a conditioned media

** See Table S6 Materials and reagents for production of Rspondin1 conditioned media

***The organoid growth factors are reconstituted in supplemented basal media. A 83-01 is reconstituted in DMSO.

Volumes can be adjusted to suitable amounts.

6. **Murine model of PDAC with age-matched control preparations**

Mice (C57/BL6, 5-8 weeks old, male, n=6) were used. The mice were housed in a temperature-controlled environment with a 12-hour light/dark cycle and provided with food and water *ad libitum.* Mice were monitored daily for health and well-being, and euthanasia was performed using CO_2 inhalation followed by cervical dislocation, as recommended by the Norwegian Veterinary Association (NVF) guidelines.

- Cotton swabs (sterile)
- 1 ml syringe
- 30G needle
- Inverted microscope
- Heating chamber for animal recover
- Tweezer (autoclaved/baked)
- Forceps (autoclaved/baked)
- Incubator of 37°C and 5% CO_2
- Sterile PBS
- Nitrogen liquid
- Isopentenyl
- Weight
- Fixative

Procedure of PDAC cell transplantation

1. When cells are in the exponential phase (no more than 70% confluent), collect cells from culturing flask and transfer to 15 mL tubes.

2. Spin down cells at 10,000-1,600 rpm at room temperature for 5 minutes and aspirate the supernatant.
3. Wash cells by adding 3 mL sterile dPBS and centrifuge at 1,000-1,600 rpm at room temperature for 5 minutes, and then carefully remove the supernatant.
4. Gently resuspend the cell pellet in sterile dPBS, adjusting the volume based on the required number of injections, and proceed to evaluate cell viability, quantity, and concentration.
5. If cell preparation occurs away from the animal facility, ensure their transport on ice to preserve viability.
6. After verifying cellular viability, incubate the cells in an incubator with 5% CO_2 and 37°C for 5-15 minutes.
7. Gently mix cells well before injection
8. Mice are injected with 100 µl cell suspension containing 250 000 cells.
9. The mice should be carefully monitored daily in compliance with the Animal Welfare Act and the FOTS application guidelines. Body weight must be measured daily. The first mouse was euthanized 9 days post-injection and the last after 21 days (Fig. S5).

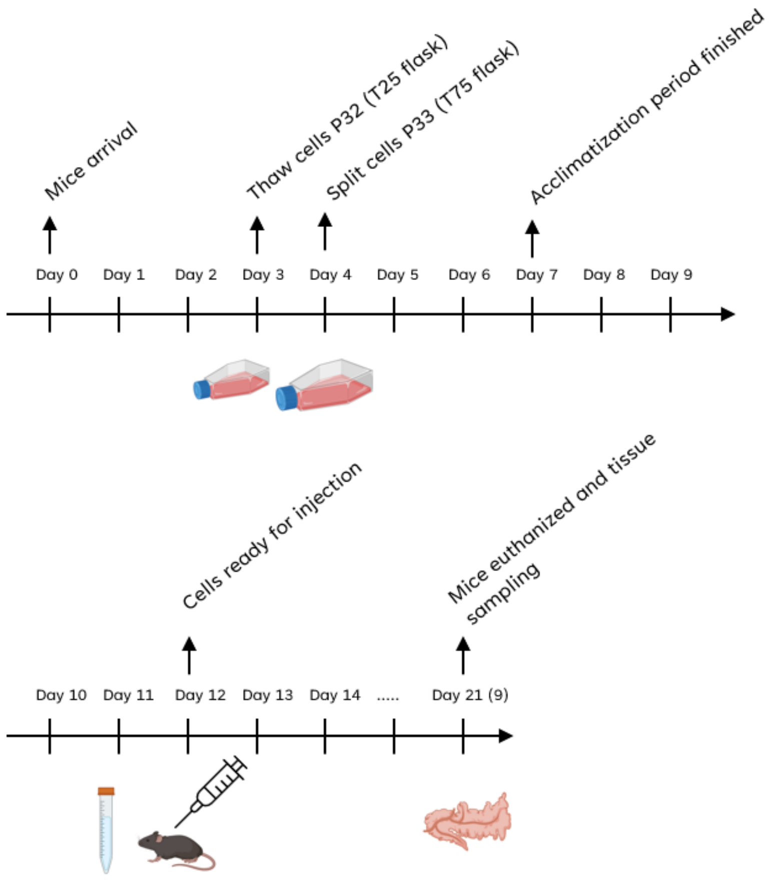

Fig. S5 Timeline of PDAC mouse model generation.

Harvesting pancreatic tissue of mice: All surgical procedures were conducted under isoflurane inhalation anesthesia at a concentration of 2-3%, and postoperative analgesia was provided using 0.1 mg/kg buprenorphine administered subcutaneously.

1. Weigh mouse before surgery.
2. Place mouse in the anesthesia chamber and transferred to operation table when properly anaesthetized.
3. Euthanize mouse by cervical dislocation. Open the abdomen and retrieve the pancreas.
4. Collect tissue samples and place them with snap freeze and keep frozen at -80°C until further processing.

7. Proteomics procedure

Cells, spheroids, tissue samples, and organoids are prepared in a similar manner, with the exception of certain tissue samples that require homogenization prior to further processing.

The preparation of these research models is crucial for ensuring accurate proteomic analysis by removing any contaminants and growth media. For organoids, it is particularly important to eliminate the Matrigel matrix, which can interfere with downstream proteomic procedures. To achieve this, follow the procedure outlined in "Sample Preparation of Organoids." After step 5. when the Matrigel is removed, snap freeze the organoids.

Sample preparation of cells and spheroids:

Cells and spheroids do not contain Matrigel and can be prepared accordingly:

1. Spin at 500 x g at room temperature for 5 minutes.
2. Discard medium and add PBS.
3. Spin at 500 x g at room temperature for 5 minutes.
4. Repeat step 1-3 three more times.
5. Snap freeze in liquid nitrogen and store at -80°C until ready for proteomics analysis.

Sample preparation of organoids

1. Remove the medium from the 24-well plate and add 1 mL of cold Cell Recovery Solution. In cases where the Matrigel is loose, carefully withdraw 400 µL from the surface, then add 400 µL of recovery buffer directly into the well.
2. Use a Pasteur pipette pre-rinsed with precooled Anti Adherence Rinsing Solution to gently triturate the dome twice using the pipette tip. Subsequently, transfer the organoids into a 15 mL pre-rinsed with Anti-Adherence Solution. This method of breaking the dome into smaller fragments facilitates a more effective digestion of Matrigel.
3. Position the 15 mL tube horizontally on ice within an ice box, then place the box on a rotating or tilting platform to gently agitate the suspension. Completion of incubation is indicated by the dissolution of Matrigel and the organoids beginning to float in suspension, which may necessitate an incubation period extending one hour.
4. Allow the organoids to settle at the bottom of the conical tube by gravity for 15-20 minutes at room temperature.
5. Carefully remove the supernatant, which will primarily contain the Matrigel. If necessary, spin at 50-100 x g for 5 minutes at 4°C.
6. Remove the supernatant, snap freeze the organoids in liquid nitrogen and store at -80 °C.

Preparation for mass spectrometry (MS):

1. Add 100 µl 1% SDC, 100mM Tris-Hcl pH-8.5, 10mM TCEP, 40mM CAA to each sample.
2. Place on a thermoblock at 95°C for 10 minutes.
3. Transfer the solution to sonication tubes and sonicate 30 sec ON/30 sec OFF x 10.

4. Measure the protein concentration.
5. Double the volume with 0.1M Ammonium bicarbonate
6. Add 1ug of trypsin to the same tube.
7. Place on termoblock at 37°C, 800 rpm overnight.
8. The next day, add 10% TFA to pH~2 to all samples.
9. Spin at 5000 rpm at room temperature for 1 minute.
10. Desalt the peptides using C18 matrix. In-house made spin columns or commercial.
11. Add 50µl CH_3OH to the chromatography columns and spin at 5000 rpm for 1 minute.
12. Add 50 µl 0,5 0,1%TFA and spin at 5000 rpm for 1 minute.
13. Add peptide mix and spin at 5000 rpm for 1 minute and add another 50 µl 0.5% TFA and spin at 5000 rpm for 1 minute.
14. For elution add 50ul of 80% CH_3CN, 0,1% TFA and spin at 5000 rpm for 1 minute.
15. SpeedVac until dry.
16. Resuspend in 20 µl 0,1% FA.
17. Spin and transfer to MS tubes.

MaxQuant was used to analyze the mass spectrometry (MS) data by identifying and quantifying proteins in the biological samples. The peptides are identified with the Andromeda search engine and quantifies proteins using label-free quantification (LFQ). MaxLFQ, a feature of MaxQuant, uses advanced algorithms to determine protein abundances by extracting and normalizing peptide signals across multiple samples. The software aligns retention times and matches features between runs, ensuring accurate and robust protein quantification. MaxQuant's user-friendly interface and integration with statistical tools make it ideal for proteomics research[6].

This comprehensive proteomic data can be leveraged in multiple ways: identifying new drug targets by analyzing crucial proteins and signaling pathways in PDAC progression, understanding disease mechanisms, discovering biomarkers for early detection and treatment monitoring, developing personalized treatment strategies, designing combination therapies that target multiple aspects of PDAC, and validating the representability of various research models.

Moreover, the data allow for a detailed comparison of the similarities and differences between the models and human PDAC tissue, identifying potential mismatches. This comparison can highlight which methods most closely resemble human PDAC tissue, guiding future research and enhancing the development of more effective PDAC therapies. By making this data publicly accessible, the research community can collectively enhance the understanding and treatment of PDAC.

8. Electron microscopy procedure

UN-KC 6141: Fixed in 2.5% glutaraldehyde in phosphate buffer. Infiltrated in 4% gelatin, and one is osmicated in regular 2% osmium in phosphate buffer, the other in potassium ferrocyanide and osmium in cacodylate buffer. The cells were infiltrated in 4% gelatin, fixed in 2.5% glutaraldehyde in 0.1M phosphate buffer pH 7.4. One sample cell was osmicated in 2% osmium in phosphate buffer, the other was in potassium ferrocyanide and osmium in 0.15 M cacodylate buffer pH 7.4. The cells were post-fixed and dehydrated with gradient ethanol and embedded in Epoxy resin. The semithin sections at 700nm were precisely cut by Leica UC7 ultramicrotome, placed on microscope slides, stained with toluidine blue O, and examined under a light microscope to assess structural details. Ultra-thin sections were cut at 60 nm, mounted on formvar-coated 50 mesh copper grids and stained with 4% alcoholic uranyl acetate and 1% lead

citrate to enhance contrast. After rinsing and drying, the sections were analyzed using a Technai 12 transmission electron microscope at 80kV. Images were captured using a Morada digital camera with Radius software, allowing for detailed visualization and data capture.

Spheroids: Followed by rinse in the same buffer. The fixative was meticulously applied, drop by drop, to preserve the structural integrity of the spheroids and then post- fixed in a mixture of 2% osmium tetroxide and 1,5% potassium ferrocyanide (KFC) in phosphate buffer, then rinsed again in phosphate buffer. The spheroids were post-fixed and dehydrated with gradient ethanol and embedded in Epoxy resin. The semithin sections and ultrathin sections were cut, contrasted and examined by Technai 12 transmission electron microscope as cells. The spheroids used for SEM purpose were dehydrated through a gradient ethanol series, followed by dehydration in acetone, and then underwent critical point drying using a Leica CPD 300. They were mounted on SEM pins with carbon tape and sputter-coated with 30 nm layer of gold/palladium using a Leica ACE 600. Imaging was performed using a Teneo Volumscope SEM equipped with ana ETD detector. Imaging parameters included a 15 kV accelerating voltage, a beam current of 0,1 nA, and working distance of 13 nm.

Organoids – Mouse exocrine pancreas, mouse PDAC and human PDAC: For electron microscopy studies of organoids, the removal of Matrigel is imperative to ensure high-resolution imaging and optimal contrast. This step eliminates interference during critical preparatory processes such as fixation and staining, thereby preserving the ultrastructural integrity of the organoids for detailed analysis.

Organoids must exceed 50 µm in diameter for electron microscopy to provide sufficient depth for detailed ultrastructural analysis and to capture complex cellular interactions and organization.

1. Removing the medium from the 24-well plate and add 1 mL of cold Cell Recovery Solution. In cases where the Matrigel was loose, carefully withdraw 400 µL from the surface, then added 400 µL of recovery buffer directly into the well.
2. Using a Pasteur pipette pre-rinsed with precooled Anti Adherence Rinsing Solution to gently triturate the dome twice using the pipette tip. Subsequently, the organoids were transferred into a 15 mL pre-rinsed with Anti-Adherence Solution. This method of breaking the dome into smaller fragments facilitated effective digestion of Matrigel.
3. Positioning the 15 mL tube horizontally on ice within an ice box. Then the box was placed on a rotating or tilting platform to gently agitate the suspension. Completion of incubation was indicated by the dissolution of Matrigel and the organoids beginning to float in suspension, which might necessitate an incubation period extending one hour. When there was still Matrigel left, add additional cell recovery solution and carefully pipette the pellet with a Pasteur pipette were performed to further loosen the Matrigel.
4. Allowing the organoids to settle at the bottom of the conical tube by gravity for 15-20 minutes at room temperature or spin at 50-100 x g for 5 minutes at 4°C.
5. Carefully removing the supernatant, which contained the Matrigel, and then gently swirled the tube to resuspend the organoid pellet in fixative without pipetting up and down and added 3mL 2.5 % glutaraldehyde + 4 % paraformaldehyde in 0.15 M cacodylate buffer (pH 7.4) per 15 mL tube.
6. Fixing the organoids for at least 4 hours or overnight at room temperature.
7. Organoids were post-fixed in 2 % OsO_4 + 1.5 % KFC in cacodylate buffer and then infiltrated in 12 % gelatin in 0.1 M Phosphate buffer and prepared for TEM and SEM as described as for cells and spheroids (see attachment for detail protocols).

Age match control mice and PDAC mice: After tissue removal, it is crucial to treat the tissue quickly and correctly by considering factors such as transport, fixation method, time, temperature, and pH to preserve structures, prevent autolysis, and ensure research viability, using 10-20x the liquid volume of the tissue sample, as improper fixation can degrade structures and affect antibody recognition.

1. Removing a small piece of pancreatic tissue from the animals, put it on a wax plate and add a small volume of primary fixative (2.5% glutaraldehyde + 4% paraformaldehyde in 0.15M Cacodylate buffer pH 7.4) on the tissue to keep it moist.
2. Cutting the tissue into cubes with a double edge razor blade (not scalpel). The cubes should be 1x1x1 mm in size (for optimal penetration of fixative). It was important not to dry out the pieces and keep them in the same fixative at all the time. Place the sample pieces into a vial containing enough fixative to cover the tissue well. Make sure that the pieces were immersed in the solution.
3. The tissues were fixed, rinsed twice and then transferred to 2,5% glutaraldehyde for storage prior to embedding at 4 °C.
4. Secondary fixation was performed by incubation for one hour in a mixture of 2% osmium tetroxide and 1.5% potassium ferrocyanide, prepared in 0.15M cacodylate buffer at pH 7.4.

The dehydration, embedding, sectioning were described as earlier (see attachment for detail). In briefly, the samples began on a shaker with gradient ethanol, embedded in resin and preparing the samples for subsequent sectioning and analysis under a microscope as described earlier (see attachment for detail protocol).

Human tissue: The tissue samples went through a standard protocol including fixation/stabilization, dehydration, embedding, sectioning, and contrasting.

Procedure:

Initially, primary fixation is performed using 2.0% glutaraldehyde in cacodylate buffer at pH 7.4, maintained at about +4°C until further processing. If the sample is processed on the day of receipt, it must first stand for at least 2 hours at room temperature. The samples are then rinsed twice briefly with 0.15M cacodylate buffer at room temperature.

1. Post-fixation involves 2% osmium tetroxide in 0.075M cacodylate buffer for 2 hours in a cooling environment maintained similarly at around +4°C, followed by another rinsing phase with cacodylate buffer at room temperature.
2. The dehydration and infiltration process, using a Leica EMTP (Ortomedic, Leica catalog no. 16709202), involves a series of alcohol concentrations and acetone treatments at room temperature. Specifically, the tissue goes through gradient dehydration of 50%, 70%, 90%, and absolute alcohol, each for 30 minutes. The tissue is then treated with 2% uranyl acetate in absolute alcohol for another 30 minutes, followed by two 20-minute acetone washes. The infiltration process continues with a graded series of acetone and Epon mixtures, first at a 2:1 ratio for 60 minutes, then 1:1 overnight, followed by a 1:2 ratio for another 60 minutes, and finally in pure Epon for 5-6 hours.
3. Embedding is carried out using flat embedding molds, followed by curing overnight at approximately 60°C. This method involves a range of specialized equipment and materials from Leica, including various assemblies, reagent vials, baskets, and disposable components, to ensure precise handling and optimal outcomes during the electron microscopy preparation process (see attachment for detail protocol).
4. The samples were section and examined as described earlier.

9. References

1 Torres, M. P. *et al.* Novel pancreatic cancer cell lines derived from genetically engineered mouse models of spontaneous pancreatic adenocarcinoma: applications in diagnosis and therapy. *PLoS One* **8**, e80580, doi:10.1371/journal.pone.0080580 (2013).

2 Byrne, H. M. Dissecting cancer through mathematics: from the cell to the animal model. *Nat Rev Cancer* **10**, 221-230, doi:10.1038/nrc2808 (2010).

3 Laboratory, T. *Murine and Human Organoid Protocols*, 2018).

4 Roelink, H. & Nusse, R. Expression of two members of the Wnt family during mouse development--restricted temporal and spatial patterns in the developing neural tube. *Genes Dev* **5**, 381-388, doi:10.1101/gad.5.3.381 (1991).

5 Gopalakrishnan, S. *et al.* Comprehensive protocols for culturing and molecular biological analysis of IBD patient-derived colon epithelial organoids. *Front Immunol* **14**, 1097383, doi:10.3389/fimmu.2023.1097383 (2023).

6 Cox, J. *et al.* Accurate proteome-wide label-free quantification by delayed normalization and maximal peptide ratio extraction, termed MaxLFQ. *Mol Cell Proteomics* **13**, 2513-2526, doi:10.1074/mcp.M113.031591 (2014).
